# Processes for regulating genetically modified and gene edited plants

**DOI:** 10.1080/21645698.2023.2252947

**Published:** 2023-09-10

**Authors:** John R Caradus

**Affiliations:** Grasslanz Technology Ltd, Palmerston North, New Zealand

**Keywords:** Certification, Plant variety rights, multilateral agreements, precautionary principle, risk, labeling

## Abstract

Innovation in agriculture has been essential in improving productivity of crops and forages to support a growing population, improving living standards while contributing toward maintaining environment integrity, human health, and wellbeing through provision of more nutritious, varied, and abundant food sources. A crucial part of that innovation has involved a range of techniques for both expanding and exploiting the genetic potential of plants. However, some techniques used for generating new variation for plant breeders to exploit are deemed higher risk than others despite end products of both processes at times being for all intents and purposes identical for the benefits they provide. As a result, public concerns often triggered by poor communication from innovators, resulting in mistrust and suspicion has, in turn, caused the development of a range of regulatory systems. The logic and motivations for modes of regulation used are reviewed and how the benefits from use of these technologies can be delivered more efficiently and effectively is discussed.

## Introduction

Methods for breeding plants were not regulated until the advent of so called ’transgenic’ cultivars, which were commercially released in the mid-1990s. The drivers for this regulation included fear of the unknown as a result of poor communication by the science and corporate communities, misinformation and fearmongering, a view that mankind was ‘playing with nature’, and the initial absence of economic or environmental benefit resulting from mistrust and suspicion of corporate multinationals who were developing and marketing these new developments. In addition, the implementation of the Cartagena Biosafety Protocol, which was negotiated to provide guidance to national economies for both the growth and international trade of transgenic crops has had a significant impact.

Regulation is not necessarily a bad thing. In fact, it is a positive and necessary means for protecting society, the environment, and the economy; however it is the form and management of regulation that is in question here. Regulation of genetically modified (GM) crops and foods at a national level is motivated to ensure human safety, protect the environment, avoid fraud and mislabelling, and address any public concern through providing them with confidence in the actual product or process used to deliver the product.^1^ However, the Director-General of the International Food Policy Research Institute warned that “Condemning agricultural biotechnology for its potential risks without considering the alternative risks of prolonging the human misery caused by hunger, malnutrition, and child death is as unwise and unethical as blindly pursuing this technology without the necessary biosafety”.^2^ So, there is a balance to be reached here between over regulation or method of regulation and freedom to operate and, as a result, ensure that benefits of new technologies can be realised.

Why is regulation of concern when GM crops have been widely adopted in many countries for more than two decades? One issue is that legislation for regulating GM crops was adopted in the late 1990s or early 2000s, but since then molecular biology has moved on and much of that legislation may no longer be fit for purpose and at the very least needs reviewing. While some countries have begun re-evaluating their legislation and as a result, developed a position on how to manage and regulate (or not) products from New Breeding Technologies others are yet to do this. Some countries have food safety standards which allow importation and use of GM food, so long as it is labelled, but other regulations do not permit their farmers to grow and produce GM crops, as currently occurs in New Zealand^3,4^; and Ecuador.^5^ Additionally, there are situations where GM crops can be imported for animal feed, but again, other regulations do not permit their farmers to grow and produce GM crops, as occurs in Europe.^6–12^ In the EU 107 GM foods have been approved for use, but 19 of 27 states of the EU have also voted to either fully or partially restrict the use of GM crops.^13^

The purpose of regulation is to determine:

- Whether GM technologies can be safely researched in crops both in and outside of containment;
- Whether GM crops can be grown commercially for domestic or export markets, without compromising other activities or opportunities;
- Whether GM crop seed can be imported for animal feed (noting that 70 to 90% of GM crops are consumed as feedstock by food-producing animals^9,14^);
- The tolerance threshold of seed imported that may contain GM content; and
- Whether food from GM crops can be imported and consumed with no harmful effects.

Rules for regulating these different aspects may be incorporated into different legislation and managed by different regulators within a country, as is their democratic right. For example, in New Zealand the ability to grow or import GM crops is regulated by the HSNO Act, while the ability to import GM food is regulated by the Food Standards Australia New Zealand Act 1991 (FSANZ Act).^15,16^ Likewise, in Australia, the ability to grow or import GM crops is regulated by the Gene Technology Act 2000,^17^ but the ability to import GM food is regulated by the Food Standards Australia New Zealand Act 1991 (FSANZ Act). In the European Union (EU) release and growing of GM crops is legislated under Part B of Directive 2001/18/EC (the ‘deliberate release’ directive) at a national level, while the control of commercial cultivation of GM crop plants operates at the EU-level and is legislated under Regulation 1829/2003/EC (the ‘GM food and feed regulation’).^18^ In Canada, the Canadian Food Inspection Agency is responsible for regulating the release of plants with novel traits, while Health Canada is responsible for the regulation of novel foods.^19^ In USA, there is no specific legislation for regulating GM crops and so its relies entirely on using legislation was already in existence.^20^ As early as 1984, it was decided that the Food and Drug Administration (FDA) would regulate genetic engineering products no differently that those achieved through traditional techniques. The Environmental Protection Agency (EPA) described existing and proposed new policies for regulating pesticidal and non-pesticidal microorganisms. The Department of Agriculture (USDA) stated that under its different legislative authorities it could broadly regulate genetically engineered plants and animals, and plant and animal pathogens”.^21^

The aim here is to systematically review systems being used for regulating both GM and non-GM crops and forages, to examine the logic and motivations for the modes of regulation used, and discuss how the benefits from use of these technologies can be delivered safely but more efficiently and effectively.

## Definitions

### Cisgenesis

Cisgenesis is defined as transferring a gene from the same or a closely related species.^22–27^ In Australia, Canada, and the United States of America, the legal regulation of cisgenic plants is less restrictive than in Europe, Japan, and New Zealand^28^.

### Gene or Genome Editing (GEd)

GEd refers to modifying DNA at one or more specific sites using CRISPR, Zinc Finger Nucleases, or TALENs.^29,30^ These use site-directed nuclease (SDN) technologies which can be categorized as –

- induction of single point mutations or InDels, resulting in gene disruption or deletion (SDN1),
- short insertions or editing of a few base-pairs by an external DNA-template sequence resulting in gene correction or modification (SDN2), or
- DNA insertion of longer strands (SDN-3) of allochtonous (transgenes) or autochtonous sequences (cisgenes) (SDN3).^31–34^

SDN-1 and SDN-2 type gene edits are cisgenic in nature.^35,36^ This review will not consider the use of gene editing to create gene drives.^37^

### Genetic Modification (GM)

GM is here defined as the manipulation of an organism’s genes by introducing, eliminating, or rearranging specific genes using the methods of molecular biology.^38^ A similar definition is provided by the Cartagena Protocol as any living organism that possesses a novel combination of genetic material obtained through the use of modern biotechnology”^39^

### New Breeding Technologies (NBT)

NBT or New Genomic Techniques include genome or gene editing (GEd); introducing targeted changes to a small number of bases of DNA using oligonucleotide-directed mutagenesis; cisgenesis; intragenesis (inserting a reorganised regulatory coding region of a gene from the same species); RNA interference for gene silencing^40,41^; and using epigenetic processes to change the activity of genes without changing a DNA sequence.^32,42,43^

### Epigenetic Modifications

Epigenetic modifications have been defined as “the structural adaptation of chromosomal regions so as to register, signal or perpetuate altered activity states”.^44^ Further Bird concluded that “without such epigenetic mechanisms, hard-won changes in genetic programming could be dissipated and lost; transient disruptions of chromosomal organization might go uncompensated; and DNA damage might escape repair”. There is a view that epigenetically modified organisms are not currently covered by the European GMO legislation.^45^ However, through the consideration of the possible use of epigenetic modification in future breeding its definition is included here for completeness. This development also demonstrates that methods of genetic modification are moving and developing at a rate that current legislation for their regulation maybe outdated and not fit for purpose.

## Motivations for Regulating GM Crops, Foods, and Feeds

Many and varied authorities and influences have motivated the need for and means of regulating GM crops, food (for humans), and feed (for animals). This has included the impact of existing regulation systems for non-GM seed and crops, societal reflections both positive and negatively toward GM crops, national regulation of GM technologies, plus a range of multi-lateral agreements, and the views of world trade and economic organisations. Regulation of non-GM crops can be focused on non-safety assessment to determine whether a new cultivar adds value over existing cultivars. It has been argued this non-safety assessment should be extend to GM and gene edited crops.^46^

### Regulation of Non-GM Seed and Crops

#### Seed Certification

Regulating seed products through registration or certification has been a feature in many countries to avoid consumers being misled and/or sold the wrong or poor quality seed.^47^ Seed certification systems are based on the product and how it is multiplied and grown.^48,49^ It has been observed that even without the regulations used to manage GM crops “seed is one of the most highly regulated commodities in the world … . and in most countries of the world, various—and sometimes multiple—government agencies are vested with supervisory and/or regulatory powers, which become levers of control”.^50^ The OECD Scheme for Certification is an international recognized program, established in 1958, with 61 participating countries, and promotes the use of certified agriculture seed that is of consistently high quality across 204 agricultural and vegetable species.^51^ The full list of cultivars across all species eligible for Seed Certification under the OECD scheme can be found at.^52^ In North America, the basic principles of certification were developed in the first half of the Twentieth Century and the Association of Official Seed Certifying Agencies (AOSCA) Advisory Board was established in 1969/70 with the primary purpose to review and approve genetic purity standards.^49^

The European Seed Certification Agencies Association (ESCAA) brings together all seed certification bodies from European Economic Area (EEA) and European Free Trade Association (EFTA).^53^ The main objectives of ESCAA are to improve communication between European seed certification agencies, the exchange of experiences associated with national seed certification systems, and to harmonize the implementation of EU legislation.

In Sub-Saharan Africa, several policy making bodies focus on seed policy harmonization, including the Common Market for Eastern and Southern Africa (COMESA, Lusaka, Zambia). Harmonized Seed Regulations were adopted in 2014; the Economic Community of West African States (ECOWAS, Abuja, Nigeria) Seed Regulations passed in 2008; and harmonized seed regulations for the Southern African Development Community (SADC, Gaborone, Botswana) were adopted in 2013.^54,55^

In New Zealand, seed certification was implemented in 1929 as a voluntary system to ensure that cultivars of important agricultural plant species maintain their identity through successive generations of multiplication for the ultimate benefit of end users.^56^

#### Plant Variety Rights

**Figure 1.**
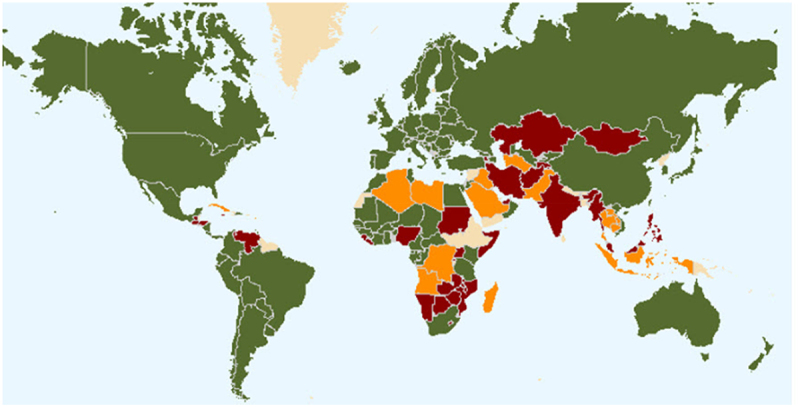
Status of UPOV membership as of September 2022. Green – members of UPOV (78)(covering 97 States); Red – initiating states (19) and organisations (1); and Orange – States (23) and organisations (1) in contact with the UPOV Office.^61^.

Aligned with seed certification systems was the development of legislation for protecting a plant variety, now commonly known as Plant Variety Rights, Plant Breeders’ Rights, or Plant Variety Protection.^57,58^ The purpose of this was to encourage variety development by providing protection to those who develop new varieties, and thereby encourage the investment of private funds in plant breeding research programs.^59^ The International Union for the Protection of New Varieties of Plants (UPOV) is an intergovernmental organisation headquartered in Geneva (Switzerland) established to provide and promote an effective system of plant variety protection, with the aim of encouraging the development of new varieties of plants, for the benefit of society.^60^ The UPOV Convention was adopted in Paris in 1961, and it was revised in 1972, 1978, and 1991. Membership status of countries to UPOV is shown in Fig. 1.

To be eligible for protection through Plant Variety Rights a cultivar must be distinct, uniform, and stable (the DUS requirements), new and have an adequate variety denomination. To establish that a new variety is distinct, it is usual to compare plants of the new variety growing alongside plants of the most similar varieties.^62^ A cultivar seeking PVR must also show uniform phenotypes within the cultivar and stable phenotypes by comparing between two seed generations of the cultivar. Assuming a cultivar is phenotypically uniform and genetically stable then it only needs to be different in one physical characteristic from all other cultivars of the same species to be granted a plant variety right.

#### National Listing of Cultivars

In some jurisdictions, new cultivars cannot be marketed or sold unless they are deemed an improvement on existing cultivars when tested in National List Trials. Registration of a plant cultivar on the National List of a country essentially provides a commercial license and ensures that cultivars on the market^63^:

1. Deliver profitable results in terms of productivity;
2. Facilitates trade and provides more opportunities to farmers; and
3. Provides the user of seed guarantees with respect to varietal identity and purity, germination capacity and specific purity as well as seed that is appropriate from a plant health point of view.

This process is used in a number of countries which includes, but not exclusively, Europe and the UK,^63–67^ Norway,^68^ Canada,^69^ Ukraine, Kazakhstan and Georgia,^70^ Egypt,^71^ some Sub-Saharan African countries,^54,72^ Kenya,^73^ Zambia,^74^ Brazil,^75^ Costa Rica,^76^ Cyprus,^77^ and Malaysia.^78^

However, in many other countries, there is no requirement to register plant cultivars before being marketed and sold. This includes USA,^79^ New Zealand, and Australia. Some (e.g., New Zealand^80^ and Australia for forage cultivars^81^ and cereals^82^) do have national variety testing trials, which only rank cultivars in a range of environments but have no influence on whether they can be marketed or sold. That is a decision made by the owner and commercial producer of the cultivar, relying solely on market forces and demand to determine the extent of acceptance and uptake.

### Regulations - a Response to Societal Concerns?

The groundswell of negativity toward GM crops in the 1990s may have been an additional motivator for legislation to be established to regulate this technology. Recent surveys have shown a range of responses depending on the question asked, and the way in which it is asked,^83^ and the scientific literacy of the audience (Table 1). A recent review concluded that “attitudes towards biotech foods (either GM or gene edited) are typically driven by negative perceptions of their risk benefits and alleged unnaturalness”.^94^ Risk can be assessed by testing specific hypotheses to determine both the probability and severity of an event.^95^ Price will also impact this decision, with a lower cost for GM foods reducing the number deciding against consuming them.^86,96,97^ In an Italian survey, it was determined that students in technical and natural science programs (61% of respondents) had a better perception of GM products than those enrolled in social sciences programmes (23% of respondents).^87^ In support of this, 81% of scientist members of the Italian Association of the Agricultural Science Societies believed GM foods are safe to consume compared with 54% of the general public.^98^ In USA, less negative attitudes toward GM products were found amongst those with higher scientific knowledge scores.^99^ Amongst university based academic scientists in Ireland 79% believed there should be no immediate complete ban of all GM foods and their production.^100^ Changing perceptions and views over time also occur. In Europe, the level of concern about the use of GM ingredients in food or drinks has decreased from 63% (in 2005) to 27% (in 2019).^101^ In Canada, 40% believe that there is not significant testing on genetically engineered food to protect consumers.^102^ Perhaps, this is a legacy of their product based evaluation system where novelty of about 20% is required for testing to be required.^103^ Additionally, attitudes can be modified through providing credible information on the environmental benefits of using food from GM crops.^104–107^

**Table 1.** Recent attitudes towards consumption of food generated from genetically modified and gene edited crops.

Representative population	Question asked	Survey result	Reference
China	Consumer opinions towards allowing gene-edited and transgenic technologies	Transgenic plants − 36% agree; 34% neutral; 31% disagree Gene edited plants − 45% agree; 36% neutral; 20% disagree	^84^
	Awareness, knowledge, and opinion on GM food.	11.9%, 41.4%, and 46.7% had a positive, neutral, or negative view on GM food, respectively.	^85^
France and USA	Willingness to purchase a cisgenic apple with reduced browning	Boycott purchase: France − 42% USA − 19%	^86^
Italy (young consumers)	GM foods are safe	24% agree; 39% indifferent; 37% disagree	^87^
	GM foods are unnatural products	57% agree; 26% indifferent; 17% disagree	
	GM experimentation should be more controlled	78% agree; 17% indifferent; 5% disagree	
New Zealand	Support for gene edited food production in NZ	32% positive support, 47% neutral and 21% against	^88^
	Should governments regulate NBTs	55% strongly agree, 35% agree, 9% neutral	^89^
	Support for GM food if they contain less pesticide and better nutrition	25% support; 47% neutral; 28% against	^90^
Sweden	Attitude toward GM and plant breeding	GM: Males - positive 26%; negative 31% Females – positive 9%; negative 36% Plant breeding: Males - positive 46%; negative 6% Females – positive 27%; negative 10%	^91^
UK and Poland	Support for GM foods	19.8% positive; 52.5% neutral; 27.7% negative	^92^
USA – university students	Willingness to pay more for non-GM foods	70% unwilling; 22% willing	^93^

Attitudes toward gene editing, both in public and stakeholder acceptance was more positive than for older GM technologies.^108^ In two asynchronous online focus groups with 79 participants from Australia and New Zealand held under the auspices of FSANZ on use of New Breeding Techniques in food production found that 90% of participants agreed or strongly agreed that governments should regulate new technologies.^89^ FSANZ is undertaking a review of definitions for ‘food produced using gene technology’ and ‘gene technology’ which are deemed outdated and do not reflect the diversity of techniques now in use.^109^ This may lead to some NBT foods being excluded from the requirement for pre-market safety assessment, but it is acknowledged that there are divergent views about the acceptability and risk of NBT foods and how best to regulate them.

A significant driver of public attitude is related to the status and reliability of the information received and whether it is based on fact, opinion, or a deliberate intent to mislead. The difference between “misinformation” and “disinformation” is related to the intent with which the information is shared. Misinformation contains content that is false, misleading, or taken out of context but without any intent to deceive. Disinformation is false or misleading content purposefully created with an intent to deceive and cause harm and is motivated by the desire to influence, profit, or engender confusion (National Library of Australia).^110^ Disinformation undermines trust in business, politics, and science and contributes to an erosion of social cohesion. It has been hypothesized that Artificial Intelligence technology will make disinformation even harder to identify. A recent review of disinformation and misinformation about GM crops in social media platforms indicates that negative falsehood commentary while significant was only about 10% of the total posts.^111^ However, it was deemed to be more concerning in terms of its impact than misinformation about COVID19 and vaccines. Similarly, in a voluntary survey about the use of New Breeding Technologies in crops, it was found that the dominating factor (38% of respondents) influencing attitudes was “public confusion about food safety and health risks” of these new technologies.^112^

Misinformation/disinformation is a concern in Latin America,^113^ Africa, and particularly Kenya,^114^ and New Zealand.^115^ In USA, France and Germany surveys have shown that as “extreme opposition to and concern about genetically modified foods increases, objective knowledge about science, and genetics decreases but perceived understanding of genetically modified foods increases”.^116^

### Human Health Impacts – a Major Driver of Regulation

Understandably, food derived from any new technology needs to be rigorously tested for safety from a human health perspective when consumed.^117–120^ Determining safety of food products must be science-based, combining the identification and characterization of hazards with assessments of exposure to verify level of risk.^121^ In many jurisdictions regulations require food derived from GM crops to be tested for safety.^18,122–126^ Frameworks for regulating food safety should be science based and focus on ensuring the food is safe, healthy, and nutritious and not be complicated by politicization^121^ or opinion, which results in polarized views, confusion, and mistrust.^127^

Despite the fact that the vast majority of reviews and studies examining safety of food from GM crops show no negative impact,^128–135^ there is still scepticism due to mistrust of organisations undertaking the testing.^136,137^ Some of the studies which have indicated potential issues related to health and safety with feeding GM crops have often required more detailed analysis and commentary. The critiquing of these trials has been reviewed^29,138,139^ in an attempt to outline both sides of the debate. In all the food safety testing, the focus should be on the impact of the GM trait and not so much on the GM method used.^135,140^ Allergenicity to transgenic proteins has been used to screen out some GM crop trait (e.g., a gene from Brazil nut (*Bertholletia excelsa*) when transferred to a soybean cultivar to improve its nutritional value was shown to test positive in the serum assay for allergenicity by cross-reactivity with Brazil nut^141^). As a result, the development of that GM crop was terminated.

### Multilateral Agreements

In the 1980s and 1990s, governance of biotechnology through international consensus, coordination, and agreements was attempted by Organization for Economic Co-operation and Development (OECD), the Food and Agricultural Organization of the United Nations (FAO), the World Health Organization (WHO), and the World Trade Organization (WTO).^142^ In 1990, a report on the safety of food produced by biotechnology was viewed as a move toward international consensus on how to assess the safety of foods obtained using biotechnologies.^143^

The Cartagena Biosafety Protocol was adopted in January 2000, as a complementary agreement to the 1993 Convention on Biological Diversity and came into force in September 2003.^144^ The Protocol is a legally binding global agreement that seeks to protect biological diversity by managing the movements between countries of Live Modified Organisms resulting from the application of modern technology.^145^ It provides for a procedure to ensure countries have the necessary information to make decisions about transboundary movement, transit, handling, and use of Live Modified Organisms. Currently, there are 173 countries who have signed up to this Protocol.^146^ There is a view that the Cartagena Biosafety Protocol has entrenched structural opposition to agriculture research and innovation, and as a result, has impacted negatively on improving global food security.^147^ It has also been argued that plants with SDN1 gene edits (i.e., with no introduced DNA) may lie outside the Cartagena Protocol on Biosafety definition of a ‘living modified organism’ as ‘any living organism that possesses a novel combination of genetic material obtained through the use of modern biotechnology’.^148,149^ Interestingly, the USA, which leads the world in the development of GM and gene editing technologies, is not a signatory Cartagena Biosafety Protocol.^37,146^

Within the Convention on Biological Diversity, a multilateral environmental agreement, the Biosafety Protocol, has been negotiated to deal specifically with trade in GM crops for food and feed.^150^ The motivation for this was to protect biological diversity and to deal with threats to human health.^151^ However, this protocol has the potential to allow importing countries to impose trade barriers simply by undertaking a scientific assessment while also recognizing non-scientific factors and invoking the precautionary principle without any possibly recourse for exporters,^152^ which is in direct conflict with WTO commitments.^151^

With a membership of 189 countries, the Codex Alimentarius (Codex) Commission provides a collection of international standards, guidelines, and codes of practice for foodstuffs, including guidelines to assess “foods derived from recombinant-DNA plants, animals, or microorganisms,”^153^ so that Codex Alimentarius Members can share information on the results of Genetically Modified food safety assessments; the Food and Agriculture Organization of the United Nations maintains an online database entitled “FAO GM Foods Platform”.^154,155^ The FAO has provided guidelines for conducting food safety assessment of foods derived from recombinant-DNA plants.^156^ “It addresses safety and nutritional aspects of foods consisting of, or derived from, plants that have a history of safe use as sources of food, and that have been modified by modern biotechnology to exhibit new or altered expression of traits”. This safety assessment addresses possible toxicity, allergenicity (predominantly of proteins), compositional analysis, evaluation of metabolites, effects of food processing, effectiveness of intended nutritional modifications, and presence of accumulated toxic compounds and antibiotic-resistance genes.

Not all countries are subject to the same international obligations, which may have a bearing on how domestic regulations are used. For example, neither Canada, Australia, Chile, Russia nor the USA are bound by the Cartagena Protocol as the USA is not a party to the Protocol,^150,157^ and Canada and Australia have not ratified the agreement.^158–160^ The EU, New Zealand, China, and Japan, amongst others, have ratified the agreement.

### The Organisation for Economic Co-Operation and Development (OECD)

The OECD provides a coordinating role through the publication of consensus documents on the biology and key compositional parameters of new cultivars of crop species.^161^ The OECD has been addressing issues related to biotechnology since 1982.^162^ The OECD convenes two working parties-

1. Working Party on the Harmonisation of Regulatory Oversight in Biotechnology (WP-HROB)’ which deals with the environmental safety of genetically engineered organisms (plants, animals, micro-organisms).
2. Working Party for the Safety of Novel Foods and Feeds (WP-SNFF) addresses aspects of the safety assessment of foods and feeds derived from genetically engineered crops.

In 2018, the OECD brought together 35 countries to focus on applications of genome editing in the agricultural sector and discuss whether genome editing should be regulated like other genetic engineering/modification (GE/GM) methods.^163^ While this meeting did not intend to deliver recommendations, it did provide some useful insights on regulatory oversight:

- It should be science based and should aim to avoid conflict between the precautionary principle and the innovation principle;
- Some current regulatory requirements result from social, legal, and political constraints and not scientific rigour;
- Product-directed multi-tier risk assessment strategies are likely to be more resource efficient for both the applicant and the regulator; and
- Communication by both advocates and opponents needs to be fact and science based, without overburdening the non-specialist public with undue information.

### World Trade Organization (WTO)

In 1994, the WTO attempted to provide some common global standards for managing intellectual property rights through novelty, inventiveness, and industrial utility, and these were also to apply to biotechnology inventions^142^ but giving the right of member countries excluding them in order to maintain public order and morality.^164^ This became known as the Agreement on Trade-Related Aspects of Intellectual Property Rights (TRIPS). Developing countries were not supportive of the TRIPS agreement for not recognizing cultural, political-economic, and ecological dimensions and for pushing globalization while disadvantaging local practices.^165^

Under the auspices of the WTO Committee on Sanitary and Phytosanitary Measures, delegations from Australia, Argentina, Brazil, Canada, Dominican Republic, Guatemala, Honduras, Paraguay, USA, and Uruguay met in 2018 and signed an international statement on agricultural applications of precision biotechnology.^32,166^ While the final text of the international statement is non-binding, it provides the necessary guidelines for preventing regulatory asymmetries and, in turn, potential trade disruption.^167^ Recognizing the positive contributions of precision biotechnology to global agriculture and emphasizing the importance of early action to identify avenues to minimize the trade impacts of differing regulatory approaches the following (abridged and amongst others) was acknowledged:

- Precision biotechnology products have the potential to play a critical role in addressing the challenges facing agricultural production;
- Given the differences internationally in approaches used to assess agricultural biotechnology, due consideration should be exercised by governments to avoid arbitrary and unjustifiable distinctions between end products derived from precision biotechnology and similar end products obtained through other production methods;
- Due consideration should be given to available scientific and technical information when updating existing regulatory frameworks or applying these frameworks to products of precision biotechnology;
- Regulatory approaches necessary to help ensure safety in respect of products derived from precision biotechnology should be science- and risk-based, transparent, predictable, timely, and consistent with relevant international trade obligations;
- Collaborative work should promote constructive dialogue with trading partners and agricultural stakeholders on potential trade issues related to precision biotechnology, so as to support open and fair trade and encourage research and innovation; and
- Public communication efforts can build trust in regulatory frameworks and improve the acceptability of future agricultural innovations that will help farmers address global challenges.

However, many countries are not signatories to this 2018 Agreement despite the science-based rules from the original Agreement on the Application of Sanitary and Phytosanitary Measures (SPS), established in 1986 (the Uruguay Round) being signed in 1995 by all members of the WTO, including the European Union.^150^ Science-based Sanitary and Phytosanitary rules became politically unacceptable in some jurisdictions with the commercialization of GM crops. This has result in the uncoordinated development of regulatory regimes and trade rules for GM crops resulting in disjointed international trade processes exacerbated by zero tolerance standards for GM material.^168,169^

## GM Regulation in Major Economies (Based on GDP Ranking)

Examination of the GM regulations for the top 10 countries based on economic activity measured through Gross Domestic product (GDP) provides an indication of likely global trends, particularly if some reticent for using GM technologies is linked to concerns about trade tariffs when GM crops and forages are used. The top 10 economies represent over 66% of global GDP.^170–173^ There is some debate about the tenth placed economy with different databases providing Russia, Brazil, or South Korea as alternatives. Since Russia has a complete ban on GM crops and South Korea is a more industrial economy, Brazil has been chosen for this exercise. It is also the only country from South America in the top 10 economies. Unfortunately there is no top ten economies found in Africa (top economy is Nigeria at 26^th^) or the Middle East (top economy is Saudi Arabia at 19^th^).

A recent review of regulatory position of four of the top global economies (USA, Japan, European Union, and Canada, along with Australia and New Zealand) provides some significant insights into attitudes and approaches to using gene edited cultivars (Table 2).^4^ They indicate that the technical inability to identify or measure certain types of gene edited cultivars will make it difficult if not impossible to enforce the legislation in those countries still attempting to regulate the importation and use of gene edited crops and crop products.

**Table 2.** Number of GM plant events authorised for (a) commercial cultivation and (b) for food and/or feed use - since 1992 per jurisdiction Source^4,174^.

Application	USA	Canada	Australia	Japan	NZ	EU	Norway	Switzerland
Cultivation	184^a^	144	56	145b	0	10^c^	11d	0^e^
Food/feed	370	293	142	375	142^f^	226	11	4

a - Stacked events of registered single events are not included in the US list.

b - No commercial cultivation despite approval; Japan has approved a lot of commercial GMOs for cultivation.

c - Commercial cultivation with one event only in some regions of the Union (Spain and Portugal).

d - Only cultivation of blue carnation for decoration purposes allowed.

e - Moratorium for commercial cultivation in place since 2005.

f – Only approved for use as food (not feed).

Nigeria regulates GMOs through two agencies: The National Biotechnology Development Agency (NABDA) (https://nabda.gov.ng/) which focuses on biotechnology policy, while the National Biosafety Management Agency (NBMA) (https://nbma.gov.ng/) focuses on the biosafety regulations of biotechnology-derived products. The regulatory challenges faced by countries in Sub-Saharan Africa in the development and commercialization of GM crops has been reviewed.^175^ Most African countries are signatories to Convention on Biological Diversity and the Cartagena Protocol on Biosafety. Some countries such as Zambia and Kenya have banned the importation or use of GM crops, while others such as Uganda have invested heavily in GM technologies.^176^

Saudi Arabia allow the importation of biotechnology plant products, but they are required to be labelled if they contain more than one percent genetically engineered plant ingredients.^177^ A significant percentage of the processed foods imported almost certainly contained GM plant ingredients. Regulations allow for the import of biotechnology derived seeds, but Saudi farmers have not shown an interest in importing or planting these to date. Currently, there are no ongoing commercial development activities for GM plants in Saudi Arabia.

### USA

USA does not have a specific and separate law for regulation of GM organisms but rather uses the Coordinated Framework for Regulation of Biotechnology, 51 Fed. Reg. 23, 302 (June 26, 1986) to direct regulatory bodies to use the same health, safety, and environmental laws that also apply to conventional products.^4,21,178–180^ This can involve the Food and Drug Administration (FDA) to determine the safety of the GM product as a food, Environmental Protection Agency (EPA) to determine environmental impacts, and USDA’s Animal and Plant Health Inspection Service (APHIS), which is mandated to oversee that the introduction of GM plants do not pose a pest risk to plants and to have regulatory oversight of non-regulated GM plants for cultivation and transport.^181,182^ In 2017, the Coordinated Framework for the Regulation of Biotechnology was updated to modernize the regulatory system and to confirm the roles and responsibilities of the three principal regulatory agencies with respect to regulating biotechnology products.^183,184^ This system is more focused on characteristics of the biotechnology product itself than the process used for its development.^185^ Gene edited crops without recombinant DNA in the product, lacking plant pest or pesticidal activity and showing no food safety attributes different from those of non-GM bred crops are not regulated. However, effective 31 July 2023, the EPA has mandated that it will still require developers to submit data showing that plants that have been gene edited to resist pests will not harm other components of the wider ecosystem or cause a health risk.^182^

### China

In China, biotechnology has been specified as one of the frontier technologies to achieve food self-sufficiency.^186^ Managed by the Ministry of Agriculture regulations on Administration of Agri- cultural GMOs Safety (RAAGS) (Decree 304) apply to animals, plants, microorganisms, and their products. Three further decrees regulate agricultural GMOs for biosafety evaluation, biosafety administration for imports, and labelling. The Chinese regulatory system is pro-technology and science-based and does not recognize non-scientific objections to the commercialization of GM crops or GM products entering the food supply chain. All GM products selected for labelling must be labelled or otherwise they would be banned from being imported into, and sold, in China.

As early as 2002, 17 products, including GM soybean, maize, canola, cotton seeds, and tomatoes were approved for import if labelled. These were for processing and consumption either as food or feed or export. In 2009, the Ministry of Agriculture issued production safety certificates to two GM crops: Bt rice (Huahui-1 and Xianyou-63) and phytase maize (BVLA430101), which allows them to be released for commercial production. Production safety certificates were issued in 2019/20/21 for a further nine GM crops of maize and soybean exhibiting insect resistance or herbicide tolerant traits. However, by 2022, none of these had been grown and commercialized. This inaction appears to have been more to do with politics than science.

While the China government invests heavily in biotechnology to increase agricultural productivity and GM crops, only recently have gene edited crop been permitted for commercial cultivation.^187^ Yield of both maize and soybean in China are much lower than the global average and China therefore has become increasingly reliant on imports of both crops, much of which will be genetically modified. China is the world’s largest importer of soybean seed (60% of world imported soybean) and second largest importer of maize grain (20% of global maize production). So, while China have for many years imported GM crops, it has only recently considered producing GM crops for domestic consumption.^188^ Further, amended the regulations in 2022 regarding the administration and commercialization of GM crops and gene-edited crops was undertaken to allow for commercialization of major GM crops. This has allowed for evaluation of gene-edited crops not being subjected to the same regulations as GM crops.^186,189^

### Japan

Japan ratified the Cartagena Protocol on Biosafety and established the “Act on the Conservation and Sustainable Use of Biological Diversity through Regulations on the Use of Living Modified Organisms” (referred to as the Japanese Cartagena Act) for implementing the Cartagena Protocol.^4,190,191^ There are two steps in the development of a GM crop cultivar in Japan – laboratory research in protected containment and then experimental field testing in an isolated area. The Ministry of Agriculture, Forestry, and Fisheries and the Ministry of the Environment assess the second step and if there is clear evidence for protection of biodiversity at the isolated site then the new GM crop cultivar can be grown commercially. Safety assessment of GM foods is undertaken by the Ministry of Health, Labour and Welfare based on the Food Sanitation Act. Two tests are required – GM food should not have any significant differences in physical or nutritive characteristics and the protein generated by the GM crops should not be noxious, and cause allergies. While Japan has approved eight types of GM crops for commercial use, farmers have been reluctant to grow them for fear of consumer criticism.^192^ Despite that the amount of GM crops imported is substantial.^4^

The Food Labelling Act requires the seller to label the food irrespective of whether it is GM-free or not. GM-free food is to be kept separate from GM food throughout production and marketing.^4^ Unintentional contamination is permitted up to 5% of the total weight of the final product and still remain GM-free.

In February 2019, the Japanese government defined genome-edited end products derived by modifications of SDN-1 type (i.e., directed mutation without using a DNA sequence template) as not representing “living modified organisms” according to the Japanese Cartagena Act.^193^ In 2021, Japan was one of the first countries to approve a gene-edited tomato (named “Sicilian Rouge High GABA”) aimed at the home garden market with an increased amount of naturally occurring γ-aminobutyric acid providing purported benefits of relaxation and reduced blood pressure.^194,195^

### European Union (EU) Countries

Three European Union countries, Germany, France, and Italy fall within the top 10 economies and will be taken as one because they are all bound by EU GM regulation. In the mid-1980s, the European Commission sought to develop a coherent regulatory approach to GM crops technologies with the view to protecting both health and environment but also ensuring free circulation with the European Union of products originating from GM technologies.^196^ Certainly, their regulatory system has achieved the first of these objectives by ensuring preventative risk management for both human health and the environment, by restricting GM crops to be grown. The exceptions here are in Spain and Portugal who have unilaterally allowed the production of insect resistant GM maize.^197^ This had also included Czech Republic, Slovakia, and Romania until 2017 when use of GM maize in those countries ceased.^157^ Since 2012, the area planted to insect resistant GM maize in Spain is approximately 30%–35% of the total maize area.

The EU regulatory system is precautionary, process based, and has been considered by others as sceptical of science,^4,198^ allows for non-scientific objection with the potential for political interference,^199^ and described as “hopelessly messed up”^200^ and in “gridlock”.^201^ The EU regulatory system has been elsewhere summarised as ”inconsistent from the viewpoint of environmental and health risk, scientifically outdated, slow and costly, lacks conceptual clarity, and hampers scientific and technological development”.^202^ With regard to regulation of New breeding Technologies, on 25 July 2018, the Court of Justice of the European Union declared that “organisms obtained by means of techniques/methods of mutagenesis constitute GMOs within the meaning of that provision” and “only organisms obtained by means of techniques/methods of mutagenesis, which have conventionally been used in a number of applications and have a long safety record are excluded from the scope of that directive” under the directive 2001/18/EC.^203,204^ However, there is a chance of new legislation being proposed to provide CRISPR-edited plants with a regulatory framework, but it is considered that the proposed legislation will not be the best possible, even if it is passed due to the constraining influence of the current GM regulatory framework.^64^ And there are other views that propose that for genome editing applications, the level of robustness in the evidence currently required for the Environmental Risk Assessment of GMOs needs to be maintained.^205^

The drivers for the very different approaches to risk management through regulation by the EU and USA has been argued to have resulted from “a cultural struggle over both the values associated with rational science and regulatory trust” in the EU.^206^ Indeed, it has been argued that “development of EU regulation of GM crops were shaped by antecedent events, notably bovine spongiform encephalopathy (BSE) or ‘mad cow disease’ and the public fears that ensued around food safety”.^207^ However, in the USA, “vigorous promotion of GM agricultural exports during WTO meetings revealed a subjective management of free markets and not the objective science of public health and protection of the environment”^206^deduced by reference to.^208^

While much of Europe (other than in small areas of Spain and Portugal^197^) restricts the planting of GM crops, it does rely heavily on GM technology but from outsourced production.^209–212^ The majority of their pigs, chickens, and to a lesser extent cattle rely on the importation of 15M metric tons of soybean meal annually from North and South America^11^ (90% of which is GM). In 2022, the European Food Safety Authority (EFSA) provided an assessment of the safety of herbicide tolerant GM oilseed rape, cotton, and soybeans crops and also renewed the authorization for GM cotton used for food and animal feed concluding that they are as safe as their conventional counterparts with respect to the potential effects on human and animal health and the environment.^213^ The European Commission publishes a register of GM approvals which includes many for import, processing, food and feed applications, but sparingly for cultivation.^214^

In July 2023, the EU published a proposal for a Regulation of the European Parliament and Of the Council on plants obtained by certain new genomic techniques and their food and feed and amending Regulation (EU) 2017/625.^215,216^ The New Genomic Techniques (NGTs) include “more targeted and precise modifications to the genome than conventional breeding or established genomic techniques, and these modifications could or could not be produced in nature or obtained by conventional breeding techniques”.^217^ This reflects an exciting time of change in how some aspects of genetic modification are to be regulated in the future.

### India

The Indian GM Crops Release Regulation has been described as one of the most regulated GM technologies in the world.^218^ In India, GM technologies include techniques by which ‘heritable material … is inserted into [the] cell or organism’ as well as ‘the formation of new combinations of genetic material by incorporation of a cell into a host cell’, and the ‘modification of an organism or a cell by deletion and removal of parts of the heritable material’.^219^ This inevitably covers all New Breeding Technologies. In 2021, while India had over 10 million ha of commercial GM crops it had not approved cultivation of any gene edited crops.

The process or product of genetic engineering technology is regulated under biosafety regulatory framework established under “Manufacture, use, import, export and storage of hazardous microorganisms/genetically engineered organisms or cells, Rules 1989^220^ under Environment (Protection) Act (EPA), 1986”.^221^ Six agencies under authority of the Ministry of Environment, Forest and Climate Change, in close collaboration with State Governments and the Department of Biotechnology, oversee the development of genetically modified crops.^222^ The six agencies are: (1) RDAC: rDNA Advisory Committee; (2) IBSC: Institutional Biosafety Committee; (3) RCGM: Review Committee on Genetic Manipulation; (4) GEAC: Genetic Engineering Appraisal Committee; (5) SBCC: State Biotechnology Coordination Committee; and (6) DLC: District Level Committee.

India has set up a National Biodiversity Authority (NBA) to regulate the use of biological resources for commercial or research purposes or for the purposes of bio-survey and bio-utilization.^223^ A draft document on Genome Edited Organisms: Regulatory Framework and Guidelines for Risk Assessment was published in 2020.^221^ This summarizes the regulatory pathway for gene-edited plants, animals and human stem cells and products derived thereof and recommends that the Regulations and Guidelines for Recombinant DNA Research and Biocontainment 2017 shall be applicable for genome editing of plants and animals. Having said that there was some flexibility with regard to SDN1 gene edits which “would be assessed mainly to confirm targeted edit(s) as well as absence of any biologically significant off-target genomic changes. Also, they would be subjected to phenotypic equivalence analysis on case-by-case basis”. SDN2 gene edits “would be assessed for phenotypic equivalence and trait efficacy through appropriate contained and/or confined field trials”, while SDN3 gene edits would be subject to “all the biosafety data requirements which are prescribed in existing food and environmental safety guidelines specific for GE (GEd) cells/organisms on case-by-case basis where foreign genes are inserted”.

### United Kingdom

With the advent of Brexit, the UK is progressing ahead of the EU with the Genetic Technology (Precision Breeding) Bill being introduced in May 2022 having completed its passage through the House of Commons and after gaining Royal Assent has become an Act of Parliament.^224^ This Act exempts certain gene editing techniques from broader GM regulations. These are referred to as “precision bred organisms”, which for their release need to be approved by the advisory committee and confirmed by The Secretary of State.^225^

### Canada

Canada has not changed its regulatory system to accommodate New Breeding Technologies largely because it is a product oriented system and therefore able to manage all crop technologies irrespective of their method of development or breeding.^4,103,121^ All plant products therefore are subject to the same regulatory framework irrespective of whether they use GM or non-GM technologies,^226^ and each is adjudged on a case-by-case basis to determine if the product is a ‘plant with novel traits’.^178^ While there is no clear definition of novelty it is generally accepted that a 20% difference in the target trait would qualify. Plants deemed to have novel traits will be tested for allergenicity, toxicity and impact on non-target organisms.

### Brazil

Biosafety impacts of new technologies on the environment and human/animal health is regulated by the Brazilian National Biosafety Technical Commission (CTNBio) through bylaw (No. 11.105/2005).^178^ New Breeding Technologies are regulated under the Normative Resolution No. 16 (NR 16) published on 15 January 2018 and indicates that techniques leading to a product not classified as a GMO are: early flowering, seed production technologies, reverse breeding, RNA-dependent DNA methylation, site-directed mutagenesis (SDN), oligonucleotides directed mutagenesis (ODM), agroinfiltration/agroinfection, topical or systemic use of RNAi, and viral vectors.^5^

For further reviews on GM regulation in different countries refer to^227^and.^228^

## Potential Impacts of Government Regulatory Systems on Innovation and Downstream Benefits

Government management of regulatory systems should be aimed at maximizing benefits and minimizing risk. Regulations can allow the safe use of innovations associated with genetically modified crops and thereby provide benefit or they can stifle innovation and deprive whole populations of potential improvements from both economic and environmental perspectives.

### Influence of Method of Regulating GM Crops

Regulatory systems have been categorised as largely either focused on the process used, or on the traits of the product developed. Interestingly, the impact of regulatory system on the number of countries with more than 0.1 million ha of commercial GM groups was similar to the number of countries with less than 0.1 million ha of commercial GM groups (Table 3). However, the underlying issue here is public trust in regulatory systems and likely confusion about the value and appropriateness of either system.

**Table 3.** Categorization of 30 countries by the regulatory system used (product versus process) and the amount of commercially cultivated GM crops. (Adapted from^159^).

Level of GM cultivation	Regulatory system	
	Product-based	Process-based
Greater than 0.1 million ha	8 – USA, Argentina, Canada, Uruguay, Philippines, Mexico, Colombia, Sudan	9 – Brazil, India, China, Pakistan, South Africa, Bolivia, Australia, Burkina Faso, Spain
Less than 0.1 million ha	6 – Honduras, Costa Rica, Bangladesh, Japan, South Korea, Russa	7 – Portugal, Slovakia, Romania, European Union, UK, New Zealand

### Influence of European Union Legislation on Use Genetically Modified Crops

The European attitude toward GM crops has had significant influence on the acceptance and uptake of this technology in some developing countries, particularly in Africa.^147,201,229,230^ Despite the fact that about two-thirds of African farmers are poor and can only benefit from new technologies that have the potential to boost crop production, some governments have adopted the European regulatory approached and driven GM food and crops from their economies.^231^ Challenges in development of biosafety regulatory frameworks and the role of individual stakeholders in the facilitation of GM crops across African countries has encouraged a centralised approach to risk assessment similar to the European Union model of the European Food Safety Authority (EFSA).^232^

An analysis of European Union GM legislation (mainly the ‘‘Release Directive’’, 2001/18/EC) using five criteria (legal certainty, non-discrimination, proportionality, scientific adaptability, and inclusion of non-safety considerations), concluded that the European regulatory framework does not satisfy the criteria of legal certainty, non-discrimination, and scientific adaptability.^233^ Others have concluded that the European Union (EU) has largely failed to create a regulatory and policy environment regarding genetically modified (GM) crops and their cultivation that is (a) efficient, (b) predicable, (c) accountable, (d) durable, or (e) interjurisdictionally aligned.^234^

### Golden Rice

Levels of pro-vitamin A in rice (*Oryza sativa*) can be deficient and result in blindness and reduced life expectancy in regions where rice is a significant part of the diet.^235^ Over two decades ago it was shown that rice could be genetically modified to elevate the expression of pro-vitamin A.^236–240^ This genetic modification became commonly known as Golden Rice. Initially criticism was that while elevated the levels of pro-vitamin A being expressed were still too low to make a real difference.^241^ This has however been corrected with levels reaching 35 μg β-carotene per gram, which can then be effectively converted to vitamin A in humans.^242^ In the last year, or so, Golden Rice became commercially available in the Philippines^243^ with 100 T harvested but was then stopped by an appeal from Greenpeace to the Philippine Supreme Court.^244^ Cultivation is still not available to all other populations suffering from vitamin A deficiency^245,246^ due to regulatory hurdles, but processes are underway in China, India, Bangladesh, Indonesia, and Vietnam to approve it.^244^

### Gene‐Edited Banana with Resistance Against Fusarium oxysporum F.Sp. Cubense Tropical Race 4

*Fusarium oxysporum* f. sp. *cubense* tropical race 4 (Foc TR4), the causal agent of Fusarium wilt of banana, has been projected to reach 17% of the global banana-growing area by 2040 equalling 36 million tons of production worth over US$10 billion.^247^ A modelling exercise suggests that regulatory delay can significantly reduce gains from a new technology for both society and industry.^248^ This study modelled the impact of 4 scenarios compared with a ‘no adoption’ scenario: (1) Immediate adoption of gene edited solution with 100% adoption ceiling reached in 15 years; (2) Late Adoption, which incorporates a 10‐year delay but with 100% adoption ceiling reached in 15 years; (3) Late‐Modest Adoption, which incorporates a 10‐year delay but with only 40% adoption ceiling reached in 15 years; and (4) Exporter delay where major exporters do not adopt the new technology and there is a 12‐year delay. It has been estimated that without any solution to *Fusarium oxysporum* f.sp. *cubense* the global discounted loss over a 40-year period would be close to US$500 billion. It is clear that with both a delay and degree of uptake that the economic impact is substantial (Table 4). the authors of the work conclude that “policy makers must recognize that there is a social cost to regulatory requirements or lack of investments in research that delays any technological introduction”.

**Table 4.** Difference between discounted aggregate consumer and producer surplus (US$ billion) over 40 years for four scenarios of adoption of a gene edited solution providing resistance against an emerging plant disease, *Fusarium oxysporum* f.Sp. *cubense* Tropical race 4 compared with no adoption of a solution under moderate disease incidence (adapted from.^248^

	Scenario			
	1. Immediate adoption	2. Late adoption	3. Late-modest adoption	4. Exporter delay
Consumer surplus change	+13.5	+7.8	+3.1	+1.4
Producer surplus change	+23.7	+13.9	+5.4	+2.1
World total change	+422	+244	+85	+44

An earlier study of using genetic modification to control *Xanthomonas* wilt disease in banana and its impact in the Great Lakes Region of Africa indicated that aggregate benefits vary across the target countries from US$ 20 million to 953 million, with the highest in countries where disease incidence and production losses are high, ranging from 51 to 83% of production.^249^ For vegetatively propagated crops, such as banana, the option of genetic modification to bring in traits of value is a potential game changer.

## Cost of Deregulation versus Benefits from the Technology

The financial and environmental benefits of GM crops have been regularly reviewed^138,139,250^ with global estimates of economic benefits being in the tens of billions of dollars per year^251^and^252,253;^ .^254^ Estimates of the costs of developing, deregulating, and releasing GM crops have been less frequently documented. Costs will be influenced by the complexity of the GM trait, the regulatory system used, and the type of organisation undertaking the work. For example, costs associated with two not-for-profit institutions’ developing one potato cultivar with late blight resistance to be made available to resource-poor farmers in a developing country was estimated to be US$1.3–1.5 million, over eight to nine years.^255^ In comparison, private sector assessments across six companies estimated that the cost of discovery, development and authorisation of a GM trait was calculated to be US$136 million, of which US$35 million was associated with regulatory requirements.^256^ Another study based on reviews and analyses of dossiers submitted to regulatory agencies and firm-level data on associated expenses calculated a range of compliance costs for developing insect resistant or herbicide tolerant maize of between US$6 and US$16 million.^257^

In Europe, it has been estimated that the cost of the regulatory process for commercial release of a new GM cultivar is between €10 and €20 million, which would be prohibitive for small and medium sized enterprises and the public sector, leaving their development to large multinational companies.^18,257^

In New Zealand, the costs associated with deregulating a GM product for commercial release has not been undertaken but similar regulations are used for the introduction of new organisms into the country. This has been estimated to cost between NZ$138, 000 and NZ$365, 000.^258^

Comparison of GM and gene-edited crops has demonstrated that the economics of gene edited crop development requires a substantially smaller market size (96.3% smaller in potential crop area) when compared to a GM crop with the same trait value and commercialization profile.^259^ This being the case then using New Breeding Techniques will become more attractive from a cost viewpoint and ensure that technology developers can focus more on output and consumer traits, rather than input traits which have dominated GM crop developments. This in turn will bring a focus on smaller niche crops where to date using GM technologies has proven to be too expensive for the value of the crop. Argentina, however, is an exemplar where small to medium sized enterprises and academia dominate the petitions for non-GM status of gene-edited organisms.^178^

## Differentiating Between Different Types of Genetic Manipulation

Pleas for international harmonisation of the safety assessment processes and safety data requirements for gene-edited crops and other NBTs that do not involve recombinant DNA^4,260^ are yet to be satisfied, just as they were (and to some extent still are) for GM crops.^261^ The debate on how to regulate gene-edited crops as distinct from GM crops has been ongoing for over a decade.^112,262,263^ Some countries have proactively differentiated some products developed using New Breeding Technologies, while others have consciously included them as part of their current GM legislation and others are yet to decide^264,265^ (Table 5). The European Union, New Zealand, Norway, and Switzerland have to date consciously included crops and forages developed using New Breeding Technologies under the same legislation as all other GM crops.^4,7,32,194,285^ This exposes contradictions. In Europe, importation of GM feed for animal is permitted but crops developed with GM technologies including those developed using New Breeding Technologies are regulated.^6,12^ It has been argued that in Europe where gene editing of crops were initially automatically classified as GM and regulated accordingly due to it requiring the use of recombinant nucleic acid techniques ignored the fact that “products of certain GE (GEd) techniques are in several cases identical for the benefits they provide from those developed by conventional or mutation breeding”.^266^ However, it is difficult to change the ideology in Europe.^286^ Similarly, in New Zealand, under the jurisdiction of Food Standards Australia and New Zealand importation is permitted for at least 90 GM produced foods which can then be sold and consumed if labelled while farmers are not permitted to grow GM crops and forages including those produced using New Breeding Technologies.^3^ The debate on the place of new technologies, including gene editing, in food production systems is underway^287^ but will still require engagement by industry and government leaders.

**Table 5.** New Breeding Technologies (NBT) legislation for growing GM crops and forages for 56 countries plus the European Union. Refer also to^32, 34, 159, 227, 264, 288, 289,^^158, 180, 228, 290, 291^ and^292^ for further detail.

Category	Type of differentiation	Country or region	Reference
Explicitly includes all NBT crops in existing GM legislation	None	European Union	^7, 32, 227, 266–273^
	None	New Zealand	^34, 178, 180, 227, 274, 275^
GM legislation allows some NBT crops to be considered as non-GM	Final products with no transgene do not fall under the Regulatory Framework for GMOs	Argentina	^5, 43^
	Introduced RNA that blocks gene expression (RNAi) and gene editing, without introduced templates to guide genome repair (SDN1 gene edits), would not be regulated as GMOs	Australia	^34, 178, 180, 276, 277^
	Does not regulate plant products if no foreign DNA present (SDN1 gene edits)	Brazil	^5, 180, 254^
	Established a science-based regulatory system that is flexible and capable of responding to new innovative products and technologies	Canada	^103,227^
	Plants mutated by CRISPR that do not contain any foreign DNA sequences are exempted	Chile	^5^, ^394^, ^180^, ^5^
	Plants mutated by CRISPR that do not contain any foreign DNA sequences are exempted	Colombia	^394^, ^180^
	Does not regulate plant products if no foreign DNA present (SDN1 gene edits)	Ecuador	^5, 158, 180^
	Authorization procedures for applications related tothe use of new genetic improvement techniques for applications related to the use of new genetic improvement techniques	Honduras	^5, 180^
	SDN-1 and SDN-2 gene edits free from exogenous DNA exempt from biosafety regulation	India	^34, 180, 227, 278^
	Does not regulate plant products if no foreign DNA present (SDN1 gene edits)	Israel	^394^, ^394^
	Does not regulate SDN-1 andSDN-2 edited plants. SDN-2 edited plants.	Japan	^34, 178, 227, 279^
	Insertion of 19 base pairs or less if DNA not regarded as GMO	Philippines	^34, 178, 180^
	Plants mutated by CRISPR that do not contain any foreign DNA sequences are exempted	Sweden	^203, 280^
	‘Precision bred’ plants now not regulated as GM	UK	^394^, ^225^
	Will not regulate, plants that could otherwise have been developed through traditional breeding techniques (namely SDN-1 and SDN-2 gene edits); but EPA will require information on safety	USA	^160, 181, 185, 227, 281, 282^
Moving toward a decision on how NBT crops are regulated	GMO definition encompasses genome editing. Discussion is ongoing.	Bangladesh	^394^, ^180^
	Safety evaluation of gene edited crops not subject to the same regulations as GMOs; but registration, seed production evaluation, seed business evaluation and processing regulated as GMOs	China	^34, 180, 186^
	Regional biosafety law for the Economic Community of West African States (ECOWAS) community is under revision Economic Community of West African States (ECOWAS) community is under revision	Ghana	^180^
	Discussion is ongoing	Indonesia	^394^, ^34, 180^
	Likely case-by-case: If no foreign DNA, then not regulated as GMO	Kenya	^394^, ^178, 180^
	Likely case-by-case: If no foreign DNA, then not regulated as GMO	Nigeria	^394^, ^178, 180^
	Proposal: If no foreign DNA,then not regulated as GMO then not regulated as GMO	Norway	^394,^
	Likely case-by-case: If no foreign DNA. then not regulated as GMO	Paraguay	^5;^^394,^^180^
	Gene edits with no foreign gene only require safety evaluation	Pakistan	^34, 180^
	Under consideration	South Africa	^178, 180^
	Not regulated as GMO if no foreign DNA introduced or retained in final product	South Korea	^34, 283^
	Discussion is ongoing	Switzerland	^158, 180^
	Discussion ongoing	Taiwan	^34^
	Draft regulations: Minimumassessment for SDN-1 products whereas SDN-3 products are assessed rigorously assessment for SDN-1 products whereas SDN-3 products are assessed rigorously	Thailand	^34^
	Discussion is ongoing	UK	^394,^
	Likely case-by-case: If no foreign DNA. then not regulated as GMO	Uruguay	^5;^^394^, ^180^
No specific gene editing regulations		Belize, Bolivia, Costa Rica, Dominican Republic, Egypt, Ethiopia, Guatemala, Malawi, Mexico, Peru, Russia, Sudan, Trinidad and Tobago, Uganda, Ukraine, Venezuela	^5, 158, 180, 284^
No specific GM regulations		Bhutan, Cambodia, East Timor, Laos, Myanmar, Nepal, North Korea, Papua New Guinea, Sri Lanka	^34^

While acknowledging the potential benefits of using New Breeding Technologies, there is also concern that poor governance as they are delivered for use will result in similar issues as occurred with the advent of GM crops.^112^ Six principles have been proposed to ensure that crops developed using New Breeding Technologies are more readily accepted^293^:

1. Risk avoidance and delivery of tangible societal benefits;
2. Robust, inclusive societal engagement;
3. Effective, science-based government regulation;
4. Voluntary best practices to supplement and complement regulatory oversight;
5. Transparency on gene-edited products in the environment; and
6. Inclusive access to technology and resources.

Argentina has been a true pioneer in legislation on gene editing, which has then been followed by many other nations within the South and Central American region.^178,180^ As a signatory from May 2000, Argentina follows the definition of a “living modified organism” in the United Nations Cartagena Protocol on Biosafety.^144,294^ They have a regulatory system that considers for an organism to be classified as GM it should have “a novel combination of genetic material,” which is based on changes present in the genome of the plant.^294^ Argentina’s vastly experienced National Advisory Commission on Agricultural Biotechnology (CONABIA) has had regulatory oversight of more than 2000 field trials and has approved 49 transformation events for commercialization in six crop species. Additionally, Argentina along with Brazil and some other Latin American countries now grow over 40% of the global GM crops area.^295^

In Europe, there is now a realisation that legislation of GM/gene editing technologies is outdated^4,296^ and support for gene editing in crops is gaining traction through “many EU politicians expressing support for these new crop products because they understand that without their adoption, EU agriculture would be at a severe competitive disadvantage to other countries who have deregulated new genome techniques”.^297^ However, how this position progresses while the anti-GM lobby remain steadfast in their resistance to these new technologies only time will reveal. Encouragingly, the European Commission recognized the current regulations of plant biotechnology, which are strictly process based, are not “fit for purpose” when it considers new genomic techniques.^298^ For environmental and food/feed risk assessment for RNAi (RNA interference which enables the silencing of target genes) in plants, it has been considered that “current science-based regulatory process in Europe is still applicable to RNAi plants; nevertheless, the assessment process should permit some flexibility for risk assessors to adapt and justify the case-by-case assessment of their RNAi plants”.^271^ In July 2023, the EU published an updated regulatory proposal on New Breeding Techniques for plants.^217^ This may result in the status quo of strict regulation and gene-edited crops remain classified as GM; or gene edited cultivars may meet certain sustainability requirements (linked to the EU Green Deal^299^) and be grown outside of limiting regulations; or gene-edited crops are permitted with no limits.^300^

A recent survey on behavioural intentions towards food derived using New Breeding Technologies (e.g. CRISPR gene editing) highlighted how consumers may be more interested in understanding potential benefits than being dissuaded about any possible risks.^301^ This signals that perceptions of benefits are relevant to acceptance and offers a potential point of distinction between food derived from GM and gene editing methods. Yet there is another argument that while the active ingredients of CRISPR/Cas gene editing are promoted for having a lower potential per reaction to create a hazard, a reduction in regulatory oversight of these and so called “null segregants” (products of gene technology but with no vestige of the technology after the segregation of chromosomes or deletion of insertions) could “create more harm faster, even if it creates benefits as well and that the potential for harm increases with increased use of the technique, but safety does not and regulations can control harm scaling”.^302^

A survey in Japan indicated that respondents found gene-edited vegetables more beneficial and acceptable than gene-edited livestock.^303^ Indeed the concerns about GM in animals relates not to “the genetic modification of animals *per se*, but rather about the types of modification that could be performed, for what purpose, who benefits and who pays”.^304^ However, as has occurred elsewhere“ genetically modified organisms can stand as a political antecedent to gene editing, and thus could have interfered with the formation of this new field”, but in Japan “collective frameworks grounded in epistemic nationalism facilitated the research and development of gene editing technologies”.^305^ Having said that, while Japan has approved 145 GM events for commercial production the country’s farmers have not adopted the cultivation of GM crops (Table 2).^4^

While previous publications have indicated that Kenya would most likely regulate GM crops on a case-by-case and if the plant contained no foreign DNA, then it would not regulated as GMO (Table 5) recent publicity indicates that the Attorney General has lost a bid to have High Court orders stopping the importation of genetically modified foods suspended.^306^

## Precautionary Principle versus Risk Assessment

Risk is defined as “the potential for harm” and is a fact of life, change and innovation. As a result any new development requires assessment to understand the associated risks and whether these are manageable and outweigh potential benefits.^307–309^ Risk should be assessed using scientific principles which involves integrity of knowledge, honesty, objectivity, and openness. The scientific method is defined as the systematic observation, measurement, and experimentation, and the formulation, testing, and modification of hypotheses.^310^ Potential risks or unintended consequences of GM crops have been extensively reviewed.^29,138,139,179,250^

### The Precautionary Principle and Precautionary Approaches

The Precautionary Principle is based on the maxim of “better safe than sorry”^311^ and has been defined as “imposing early preventive measures to ward off even those risks for which we have little or no basis on which to predict the future probability of harm”.^312^ However, there are many versions of the Precautionary Principle.^313^ How a country, organization or individual determines the way in which they may evaluate the likelihood of specific risks in relation to the Precautionary Principle is referred to as precautionary approach. The Precautionary Principle as conveyed in international laws is considered uncompromising and the precautionary approach can be viewed as an approach to the study of risk, not a “general customary rule of law or at least a general principle of law” (Quoted from^314^in.^315^ The difference between the principled approach to precaution and the pragmatic approach has been extensively debated with the conclusion that the principled-pragmatic distinction does not appear to hold in the realm of theory and that normative (i.e., prescriptive or regulating) judgments are paramount.^315^ It has been sensibly stated that “the precautionary principle is seen as a principle of common sense where action can be taken to protect health and the environment when decision makers are faced with potentially harmful effects but there is scientific uncertainty concerning the nature or extent of the risk”.^107^

Often the terms Precautionary Principle and precautionary approach are not differentiated as has occurred in Europe^316^ and Canada.^317^ The Cartagena Protocol on Biosafety^318^ does not use the term Precautionary Principle but considers the precautionary approach as a fundamental concept when using genetically modified organisms. Unfortunately, some have assumed that precaution means prevention,^319^ but this is the case since prevention applies with the assumption that a risk is known while in reality “all risks are uncertain” and thus the idea of a “known” risk is misleading,^313^ which then requires the concept of “degrees of precaution,” to avoid making a distinction between precaution and prevention. Indeed, science can never prove the absence of risk.^320^ Therefore, an uncompromising approach to the Precautionary Principle would inevitably result in no action ever being taken, because an assurance of absolute safety can never be given, leading to paralysis and a cessation of technological advancement^320–322^).

The “precautionary approach” to regulation has significantly impacted the use of genetically modified plants in a number of countries including the European Union and New Zealand, despite there being “little evidence of serious or irreversible damage to the widespread use of these crops in the rest of the world”.^135,157,323^ In New Zealand, the Royal Commission on Genetic Modification^324^ concluded that New Zealand should adopt a precautionary approach and keep it options open and “it would be unwise to turn our back on the potential advantages on offer, but we should proceed carefully, minimizing and managing risks”. However, 12 years after the Royal Commission, it was concluded that “little progress has been made toward developing the public policy capacity necessary to make effective strategic decisions on GM crops in New Zealand”,^325^ and even since then nothing has changed. Most African governments have taken the precautionary approach to introducing GM foods.^326^ The concern is that the Precautionary Principle can invoke preventative action to avert a potential harm even before that has been scientifically tested and is beyond dispute.^126^ This occurred with the monarch butterfly case tests were undertaken in a captive colony^327^ and which was later proven to be misleading with other field based studies showing that monarch butterfly populations are unaffected by the large-scale cultivation of Bt maize^328–334^ Indeed, some have considered the Precautionary Principle to be anti-science while risk assessment is likely to be objective and science motivated.^315^ While others have insisted that “the best elements of a precautionary approach demand good science and challenge the scientific community to improve methods used for risk assessment”.^335^ However, if the precautionary principle is to provide any meaningful guidance to regulators, then^320^ has argued that greater regulatory scrutiny ought to be required for the less well-characterized conventionally modified cultivars than for those where the molecular basis for the phenotype is understood as occurs for most GM crops.

Amongst some researchers in Europe, it has been argued that the “innovation principle” is a preferred option to the precautionary approach, so that relevant risk assessment would be designed on a case-per-case base, to enable benefiting from gene-edited products while complying with relevant risks management.^336^ The innovation principle ensures the impact of the innovation is fully assessed to ensure that the choice, design, and regulatory tools used foster innovation, rather than hinder it.^337^

### Measuring Risk and Managing Uncertainty

Risk evaluation should assess the probabilistic outcomes of discrete adverse events.^121^ Food and Agriculture Organization (FAO) describe risk assessment as “a scientifically based process consisting of the following steps: (i) hazard identification; (ii) hazard characterization; (iii) exposure assessment; and (iv) risk characterization”.^338,339^ However, it has been argued that “all risks are probabilistic and uncertain because we can never know the future with complete certainty”.^340^ Additionally, there is the concept known as countervailing risks which is prevalent in a multi-risk world^315^ such that an analysis of benefit versus risk is required to determine which is the action that provides the greatest benefit while affording the least risk. Unfortunately,“decision-making does not always side with relative knowledge over and against ignorance when imagining future consequences”.^315^

The Precautionary Principle has been criticized for simply addressing risks one at a time while risks are often multiple and trade-offs may need to be considered.^340^ This has led to the concept of “optimal precaution,” which does not seek to maximize precaution in one area at the expense of neglecting a risk in another but rather seeks to minimize overall negative consequences. So, the precautionary approach as distinct from the Precautionary Principle seems reasonable in managing risk and is indeed a moral responsibility.^341^ Technological progress is inevitable and with that a responsibility to evaluate the risks but from the perspective of seeking decisions based on fact, as currently understood, using scientific (not philosophic) knowledge.^341^

## Process Standards versus Product Standards

While a cautious approach to regulating GM crops may have been justified in the early years of GM crop commercialization, in the interim 25 years with an expanding knowledge of plant genomics it is now understood that “the genetic engineering process itself presents little potential for unexpected consequences that would not be identified or eliminated in the variety development process before commercialisation”.^342^ Regulatory frameworks should be product rather than process based so that it is the novelty of the characteristics or phenotype of new plant cultivars that are regulated.^18,343^ They argue that since “there is no evidence for intrinsic risks associated with GM, it is not useful to have a regulatory framework that is based on the premise that GM crops are more hazardous than those produced by conventionally bred plants”. Similarly, others have stated that “the time is right to gradually transition from process-based GMO regulations to product-based GMO regulations because many countries have had sufficient regulatory experience regarding conventional transgenesis” and in doing would extend this to genome-edited crops.^148^

If regulation of GM crops was comparable to and compatible with traditional breeding when similar traits and uncertainties are involved then this “would reduce costs, open transgenic-based innovations to a broader array of private and public entrepreneurs and thus facilitate the production of improved crops based on the genomics revolution in biology”.^342^ It will still be important in both GM and gene edited crops to examine for both target and off-target effects of gene silencing, manipulation, or introduction.^267^ Indeed, “each new cultivar, created via any method, should be tested, and assessed based on its traits and its unique profile of risks and benefits,^200^ not be the method by which it is produced.

This plea to move to a product based regulatory system is not just targeted at regions and countries with stringent systems such as Europe and New Zealand^32^ but has also been stressed as important for countries such as the USA,^344^ who to date has deregulated more GM crops than any other country.^218^ It is argued that this would “unleash the innovative potential of small companies and public sector universities and organizations”.^288,344^ An approach for moving from a process based to product based regulatory system has been proposed^178,345^ where the technology used is considered neutral.^202^ This was motivated by the increase in new techniques for manipulating genomes. It proposed that “the new product-based regulatory system should start with a presumption of no pre-market regulatory approval for crops and food with existing traits, recognized as safe”,^342^ but with the proviso that “specific types of products should be required to obtain a one-time pre-market approval for potential environmental or health risks based on red flags associated with the trait, novel or otherwise, rather than the process by which it was created”.^345^

## Co-Existence Regulations for GM Crops

Co-existence is a term that refers to the ability of farmers to make a practical choice between conventional, organic, or GM based (for approved GM crops) crop production, in compliance with the relevant legislation on labelling and/or purity standards.^346^ Strategies for allowing co-existence can be controversial and contentious^347–349^ but can also be unifying and possible^350^and.^348,351^

In the EU, co-existence and traceability arrangements are driven by the statutory requirements to label all food and feed products containing EU-approved GM or GM-derived material above a threshold of 0.9%.^348^ While EU member states were to set their own co-existence measures,^352^ some guiding principles were provided.^353^ However, the outcome has been criticised as restrictive and impractical and resulted in poor consensus among EU Member States for separation distances of cultivated GM crops.^348,354^ The European Coexistence Bureau (ECoB) has established Technical Working to provide Best Practice Documents Groups for growing GM maize, soybean, cotton, and potato with non-GM and organically farmed crops.^355^

In the UK, Supply Chain Initiative on Modified Agricultural Crops (SCIMAC), a group made up of industry organizations spanning the UK farm supply established in 1998 determined that “co-existence measures should be to permit consumer choice and freedom to operate whatever the production method involved”.^354^ They concluded that “practical measures to deliver co-existence between GM and non-GM crops can be managed effectively at the farm level and need not represent a significant departure from current best practice within the industry”.

Isolation distances to manage cross-fertilisation between GM and non-GM variants of the same species differ with species,^356^ from 5 to 10m for soybean,^357^ about 10m for cotton,^358,359^ 20 to 50m for maize,^360,361^ about 100m for *Lolium rigidum*,^362^ tall fescue (*Festuca arundinacea*) at 150m,^363^ 30 to 200m for canola,^364,365^ and 150 to 500m for lucerne.^366^ Pollen-mediated gene flow was measured in the Netherlands through growing the maize variety DKC3421YG containing the MON810 GM event at 25m (the distance indicated as required between GM crops and conventional crops) or 250m (the distance indicated as required between GM-crops and organic crops) from a near-isogenic non- GM cultivar.^367^ Averaged over 12 fields the pollen-mediated gene flow at 25m was 0.084% and 0.080%, over 2 years, and at 250m was 0.005% and 0.007%, over 2 years. The extremes were measure as 0.0% to 0.32%, variation caused by wind direction but even so well below the labelling threshold of 0.9% set by the EU.^348^ Others have also concluded that “flexible use of isolation distances with use of buffer rows of maize plants, may improve feasibility of implementing co-existence in commercial maize cultivation, particularly in agricultural landscapes with a scattered distribution of small fields”.^368^ However, in countries such as USA, Brazil, and Argentina with high adoption rates of GM crops, this could possibly exclude the development of the alternative non-GM culture of the same crop.^369^

## GM Tolerance Threshold for Imported Seed Used for Food and Feed, and Its Impact on Trade and Trade Agreements

Adventitious presence refers to the unintended presence of GM generated outputs in food and feed products.^346^ In the past, there have been situations where low-level—or adventitious—presence of GM material in shipments of non-GM crops has resulted in ban on further imports. A herbicide tolerant linseed/flax (*Linum usitatissimum*) cultivar, named CDC Triffid, developed in Canada^370^ was withdrawn from the market in 2001 in reaction to the EU’s concern with importing linseed contaminated with GM seed, through cross pollination.^147,371^ The EU has a zero tolerance policy against low-level co-mingling of GM material in imported non-GM crops, which potentially leads to international trade disruption, and supply chains being forced to spend more and more resources to satisfy rising levels of stringency as the science of detection improves.^168^ The moratorium imposed by the EU on seed lots, which did not have zero GM seed in a non-GM seed-lot was eventually ruled as a trade barrier in 2006 by the WTO. Even non-GM crop seed is never traded with the expectation of 100% purity. There is an accepted allowance of 0.25% for weed seeds and 0.25% for other crop varieties.^147^ It has been argued that the regulatory and trade challenges facing GM crops are likely to have a detrimental impact on improving global food security.^147^ However, devising a set of rules for trade in GM crops and foods is rarely part of multilateral trade negotiations and will require separate long and complex negotiations with often little room for compromise.^150^

## Labelling and Disclosure Requirements: Consumer’s Right to Know

It seems right that consumers should know what is in the food product they are consuming. An effective system of traceability for GM food and ingredients has been viewed positively in societal surveys.^372^ Issues that need to be assessed when considering mandatory GM labelling have been outlined by Oh and Ezezika^326^ as:

1. The consumers right to know, and freedom to choose to consume GM food or not;
2. Costs of implementing, which is largely associated with costs incurred from measures associated with segregation and identity preservation to prevent or limit mixing within the non-GM supply chain^373^;
3. Stigmatization associated with labelling indicating negative connotations;
4. Feasibility of enacting appropriate labelling, particularly in countries where informal markets are commonplace; and
5. Impact on food security and innovation if indeed costs increase as a result of labelling.

Labelling of food products with GM content is understood to be required in some form in a least 64 countries.^374^ Mandatory labelling of food derived from GM crops if present at any level in Peru,^375^ at levels above 0.9% is required in European Union and Norway,^46^ 1% for Australia, New Zealand, Japan,^13,376^ South Africa, Brazil, and China,^356,377^ 3% for South Korea^378^ and Malaysia,^379,380^ and 5% for USA and Canada.^13,356^ Most African countries are considering mandating labelling of GM foods not just as a rational move amidst the uncertainty surrounding the public health impact of GM foods but also for safeguarding their agricultural exports to European markets.^326^ However, there has been a plea that “labelling requirements would need to be realistic and not place unnecessarily onerous conditions on producers of GM-derived foods”.^277^ From a religious perspective “Islamic jurisprudence indicates that if a type of food is considered toxic to human health” labelling of GM derived foods should be introduced to ensure that consumers are aware of what he/she has purchased.^381^

The concern about mandatory labelling is that while consumers have the right to know what their food contains “consumer knowledge about new technology such as GE (GEd) is limited, they cannot often establish whether GM products spell danger or how to measure any given risk against potential benefits”.^155^ Interestingly, where labelling is required then a specified consumer benefit is essential for product uptake,^382^ because when products are labelled a positive correlation between consumer attitudes toward foods not containing GMOs and purchasing behaviour has been observed.^383^ The Codex Alimentarius Commission in 2007 failed in an attempt to produce labelling guidelines for GM products because no consensus was reached by the different countries involved.^384^

A study in the USA on how best to label genetically modified and gene edited foods in compliance with the National Bioengineered Food Disclosure Standard which came into effect on January 1st, 2022^385^ demonstrated that “a higher proportion of respondents choose a label if the Bioengineered label was disclosed using the approved symbol” compared with text disclosure methods. Interestingly, in this study, only 13% reported always looking for GM labels when purchasing food at a store. Stacking the Bioengineered label with a label that indicates the presence of gene editing, genetic modification, or both was also preferred.

## Elements of Effective Regulation

For any regulatory system to be effective it needs to be science-based, transparent and allow for public participation.^1,386^ The key components of regulation may include (1) mandatory pre-market approval; (2) established safety standards; (3) transparency; (4) public participation; (5) use of outside scientists for expert scientific advice; (6) independent agency decisions; (7) post-approval activities; and (8) enforcement authority and resources.^1^ The concept of considering risk measured by ‘precaution through experience’ is a “concept offering possible avenues beyond current regulatory standoffs by incorporating both scientific and socio-economic perspectives of risk in deepened deliberative settings”.^387^ Indeed, scientists have an important role to play here in understanding and engaging with the frameworks of technological determinism and Responsible Research and Innovation (RRI).^388^

Regulation is motivated to ensure human safety, protect the environment, avoid fraud and mislabelling, and address any public concern.^1^ Ideally the regulatory scheme used is focused on characteristics of the biotechnology product itself than the process used for its development,^185^ and there is an emphasis on the “innovation principle” rather than the “precautionary principle” so that each application is adjudged on a case-by-case basis to determine if the product is a ‘plant with novel traits’.^18,103,345^ A process-based regulatory systems results in an overly precautionary approach for delivery of GM crops and forages and should be avoided. The downside of a slow, burdensome, and stringent regulatory system is inevitably not just the loss in international competitive advantage in the development and use of GM crops^18^ but also a significant risk to food security and biodiversity as more land will continue to be required with a growing global population. Additionally, changing regulation processes and approaches could in some jurisdictions be a very longwinded process and therefore can become a deterrent in their own right to seeking change.^389^

Harmonization of GM regulations globally could be a preferred option. But perhaps the best that can be hoped for is harmonization within regions.^180^ This may be achievable in parts of Africa^54,390^ Latin America,^5^ and North America. It could be argued that the European Union is already harmonised.

## Concluding Comment

A range of systems have been devised to regulate new genetic variation irrespective of whether these are through non-GM, GM or gene editing methods. General consensus from commentators would indicate that those systems based on regulating the process of delivery of new genetic novelty are more challenging and restrictive than those regulating the risk of the end product irrespective of how it is produced. The challenge is for all regulators to ensure that processes they use are fit for purpose and effective in terms of balancing risks and benefits in a timely and cost effective manner.

While establishing product-based regulatory systems for GM technologies could be considered a fundamental requirement this is only one part of the solution for wider use and acceptance of GM crop technologies. Also required is better communication with the public and potential consumers of the technology. In developing countries the emphasis should be on “co-development of technologies with farmers, seeking out non-patented material and an acknowledgement that seeds are a single component of highly complex agroecological and production systems”.^391^ Additionally, consumer benefits of gene edited crops must be espoused by balancing the advantages, disadvantages and limits of different plant breeding techniques to provide a well-argued risk-benefit message.^121,392^ To garner better public understanding and acceptance of regulatory systems attempts should be made to harmonise or align regulatory systems globally.^150,393^ This is unlikely to happen until there is a collective proactive political will to ensure that innovation is not stifled by outdated and inflexible regulations.

## References

[1] Jaffe G. Regulating transgenic crops: a comparative analysis of different regulatory processes. Transgenic Res. 2004;13(1):5–19. doi:10.1023/B:TRAG.0000017198.80801.fb. 15070071

[2] Pinstrup-Andersen P. Biotech and the poor. The Washington Post. 1999 October 27. [Accessed 2023 Jan 13]. https://www.washingtonpost.com/archive/opinions/1999/10/27/biotech-and-the-poor/428df76b-5027-410f-a6ad-61f9ad4d8dc8/

[3] Caradus JR. Impacts of growing and utilising genetically modified crops and forages – a New Zealand perspective. New Zeal J Agr Res. 2022;65:1–30. doi:10.1080/00288233.2022.2077380.

[4] Spök A, Sprink T, Allan AC, Yamaguchi T, Dayé C. Towards social acceptability of genome-edited plants in industrialised countries? Emerging evidence from Europe, United States, Canada, Australia, New Zealand, and Japan. Front Genome Ed. 2022;4:899331. Article. doi:10.3389/fgeed.2022.899331. 36120531 PMC9473316

[5] Gatica-Arias A. The regulatory current status of plant breeding technologies in some Latin American and the Caribbean countries. Plant Cell Tiss Organ Cult. 2020;141:229–42. doi:10.1007/s11240-020-01799-1.

[6] Bawa AS, Anilakumar KR. Genetically modified foods: safety, risks and public concerns-a review. J Food Sci Technol. 2013;50:1035–46. doi:10.1007/s13197-012-0899-1. 24426015 PMC3791249

[7] Dederer HG. The challenge of regulating genetically modified organisms in the European Union: trends and issues. In: Nakanishi Y, editor. Contemporary Issues in Environmental Law. Environmental Protection in the European Union. Vol. 5. Tokyo: Springer Japan; 2016. p. 139–68. doi:10.1007/978-4-431-55435-6_8.

[8] Jiang L. Commercialization of the gene-edited crop and morality: challenges from the liberal patent law and the strict GMO law in the EU. New Genet Soc. 2020;39:191–218. doi:10.1080/14636778.2019.1686968.

[9] Lucht J, Lucht. Public acceptance of plant biotechnology and GM crops. Viruses. 2015;7:4254–81. doi:10.3390/v7082819. 26264020 PMC4576180

[10] Ritchie H, Roser M. Forests and deforestation. Published online at OurWorldInData.Org. 2021 [Accessed 2023 Apr 20]. https://ourworldindata.org/forests-and-deforestation

[11] Shahbandeh M. Import volume of soybeans worldwide in 2021/22, by country. 2022 [Accessed 20 Apr 2023]. https://www.statista.com/statistics/612422/soybeans-import-volumeworldwide-by-country/

[12] Van Eenennaam AL, Young AE. Prevalence and impacts of genetically engineered feedstuffs on livestock populations. J Anim Sci. 2014;92(10):4255–78. doi:10.2527/jas.2014-8124. 25184846

[13] Blagoevska K, Ilievska G, Jankuloski D, Dimzoska BS, Crceva R, Angeleska A The controversies of genetically modified food. In: IOP Conference Series: Earth and Environmental Science 854; 2021. 12009 Article. https://iopscience.iop.org/article/10.1088/1755-1315/854/1/012009/meta

[14] Flachowsky G, Schafft H, Meyer U. Animal feeding studies for nutritional and safety assessments of feeds from genetically modified plants: a review. J Verbr Lebensm. 2012;7:179–94. doi:10.1007/s00003-012-0777-9.

[15] FSANZ. About FSANZ. 2020 [Accessed 2023 Jan 27]. https://www.foodstandards.govt.nz/about/pages/default.aspx

[16] Mfe. 2004. Genetic modification - the New Zealand approach. Ministry for the environment, wellington, NZ. ME Number 426. ISBN: 0-478-24055-4. [Accessed 2023 Jun 5]. https://environment.govt.nz/assets/Publications/Files/genetic-modification-nz-approach.pdf

[17] OGTR. Office of the gene regulator. Legislative documents. 2022 [Accessed 2023 Jan 27]. https://www.ogtr.gov.au/about-ogtr/legislative-documents

[18] Baulcombe SD, Dunwell J, Jones J, Pickett J, Puigdomenech P. GM science update: a report to the council for science and technology. 2014 [Accessed 2023 Apr 7]. http://www.gov.uk/government/uploads/system/uploads/attachment_data/file/292174/cst-14-634a-gm-science-update.pdf

[19] Ellens KW, Levac D, Pearson C, Savoie A, Strand N, Louter J, Tibelius C. Canadian regulatory aspects of gene editing technologies. Transgenic Res. 2019;28(S2):165–68. doi:10.1007/s11248-019-00153-2. 31321700

[20] Marden E. Risk and regulation: US regulatory policy on genetically modified food and agriculture. Boston College Law Review. 2003;44:733.

[21] Office of Science and Technology Policy. Coordinated framework for regulation of biotechnology. 1986 [Accessed 2020 Jan 29].https://www.aphis.usda.gov/brs/fedregister/coordinated_framework.pdf

[22] Baima S, De Giacomo M, Giovannelli V, Ilardi V, Pietrangeli B, Rastelli V. Cisgenesis: an European Union (EU) perspective. In: Chaurasia A Kole C, editors. Cisgenic crops: safety, legal and social issues. concepts and strategies in plant sciences. Cham: Springer International Publishing; 2023. p. 159–78. doi:10.1007/978-3-031-10721-4_7.

[23] Becker G, Marcińska J, Artemel MN, Juszczyk A. Elucidation and acceptance—scientific, legal, and ethical aspects of cisgenesis in times of an alleged dwindling faith in science. In: Chaurasia A Kole C, editors. Cisgenic crops: safety, legal and social issues. Concepts and strategies in plant sciences. Cham: Springer International Publishing; 2023. p. 77–99. doi:10.1007/978-3-031-10721-4_4.

[24] Caplanova A, Sirakovova E. Economic considerations of cisgenics as a sub-set of genetically modified organisms. In: Chaurasia A Kole C, editors. Cisgenic Crops: safety, Legal and Social Issues. Concepts and strategies in plant sciences. Springer, Cham; 2023. p. 135–58. doi:10.1007/978-3-031-10721-4_6.

[25] Hefferon K. Cis genesis of crops. In: Chaurasia A Kole C, editors. Cisgenic crops: potential and prospects. Concepts and strategies in plant sciences. Cham: Springer International Publishing; 2022. p. 67–78. doi:10.1007/978-3-031-06628-3_4.

[26] Koul B. Cisgenics and crop improvement. In: Cisgenics and transgenics. Singapore: Springer Nature Singapore; 2022. p. 107–29. doi:10.1007/978-981-19-2119-3_3.

[27] Srinivas KR. Cisgenesis and cisgenic crops: need for a paradigm shift in harnessing and governance. In: Chaurasia A Kole C, editors. Cisgenic crops: safety, legal and social issues. Concepts and strategies in plant sciences. Cham: Springer International Publishing; 2023. p. 255–68. doi:10.1007/978-3-031-10721-4_11.

[28] Dayé C, Spök A, Allan AC, Yamaguchi T, Sprink T. Social acceptability of cisgenic plants: public perception, consumer preferences, and legal regulation. In: Chaurasia C Kole C, editors. Cisgenic crops: safety, legal and social issues. Cham: Springer International Publishing; 2023. p. 43–75.doi:10.1007/978-3-031-10721-43.

[29] Aziz MA, Brini F, Rouached H, Masmoudi K. Genetically engineered crops for sustainably enhanced food production systems. Front Plant Sci. 2022;13:1027828. Article. doi:10.3389/fpls.2022.1027828. 36426158 PMC9680014

[30] Rinaldo AR, Ayliffe M 2015. Gene targeting and editing in crop plants: a new era of precision opportunities. Mol Breeding 35: 40 Article. 10.1007/s11032-015-0210-z

[31] Doudna JA, Charpentier E 2014. The new frontier of genome engineering with CRISPR-Cas9. Sci 346: 1258096 Article. 10.1126/science.1258096 25430774

[32] Menz J, Modrzejewski D, Hartung F, Wilhelm R, Sprink T 2020. Genome edited crops touch the market: a view on the global development and regulatory environment. Front Plant Sci. 11: 586027 Article. 10.3389/fpls.2020.586027 33163013 PMC7581933

[33] FAO. Gene editing and agrifood systems. Food and Agriculture Organization of the United Nations, Rome; 2022. 86p. 10.4060/cc3579en

[34] Jones MGK, Fosu-Nyarko J, Iqbal S, Adeel M, Romero-Aldemita R, Arujanan M, Kasai M, Wei X, Prasetya B, Nugroho S, et al. Enabling trade in gene-edited produce in Asia and Australasia: the developing regulatory landscape and future perspectives. Plants. 2022;11(19):2538. Article. doi:10.3390/plants11192538. 36235403 PMC9571430

[35] Cabrera-Ponce JL, Barraza A, Alvarez-Venegas R. Cisgenic crops: major strategies to create cisgenic plants based on genome editing. In: In: Chaurasia A Kole C, editors. Cisgenic crops: potential and prospects. Concepts and strategies in plant sciences. Cham: Springer International Publishing; 2022. p. 213–35. doi:10.1007/978-3-031-06628-3_11.

[36] Ghose K, Yuan N, Dampanaboina L, Mendu V. Cisgenesis in the era of genome editing and modern plant biotechnology. In: Chaurasia A Kole C, editors. Cisgenic crops: potential and prospects. Concepts and strategies in plant sciences. Cham: Springer International Publishing; 2022. p. 257–79. doi:10.1007/978-3-031-06628-3_13.

[37] Gutmann A, Moreno JD. Keep CRISPR safe - regulating a genetic revolution. Foreign Affairs. 2018 [Accessed 2023 Jun 5]. https://www.foreignaffairs.com/articles/world/2018-04-16/keep-crispr-safe

[38] USDA. Agricultural biotechnology glossary. 2022 [Accessed 2022 Dec 22]. https://www.usda.gov/topics/biotechnology/biotechnology-glossary#:~:text=Genetic%20engineering%3A%20Manipulation%20of%20an,to%20as%20recombinant%20DNA%20techniques

[39] Secretariat of the Convention on Biological Diversity. Cartagena protocol on biosafety to the convention on biological diversity: text and annexes. Montreal: Secretariat of the Convention on Biological Diversity. 2000 [[Accessed 2023 May 11] https://www.cbd.int/doc/legal/cartagena-protocol-en.pdf

[40] Novina CD, Sharp PA. The RNAi revolution. Nature. 2004;430(6996):161–64. doi:10.1038/430161a. 15241403

[41] Ossowski S, Schwab R, Weigel D. Gene silencing in plants using artificial microRnas and other small RNAs. Plant J. 2008;53(4):674–90. doi:10.1111/j.1365-313X.2007.03328.x. 18269576

[42] Parisi C, Rodríguez-Cerezo E. Current and future market applications of new genomic techniques. EUR 30589 EN. Publications Office of the European Union: Luxembourg; 2021. p. 52. doi:10.2760/02472JRC123830.

[43] Whelan AI, Lema MA. Regulatory framework for gene editing and other new breeding techniques (NBTs) in Argentina. GM Crops & Food. 2015;6(4):253–65. doi:10.1080/21645698.2015.1114698. 26552666 PMC5033209

[44] Bird A. Perceptions of epigenetics. Nature. 2007;447(7143):396–98. doi:10.1038/nature05913. 17522671

[45] Faltus T. The applicability of the European GMO legislation to epigenetically modified organisms. Frontiers In Bioeng Biotechnol. 2023;11:1124131. Article. doi:10.3389/fbioe.2023.1124131. PMC10009104 36923460

[46] Myskja BK, Myhr AI. Non-safety assessments of genome-edited organisms: Should they be included in regulation? Sci Eng Ethics. 2020;26(5):2601–27. doi:10.1007/s11948-020-00222-4. 32424723 PMC7550366

[47] Misra MK, Harries A, Dadlani M. Role of seed certification in quality assurance. In: Dadlani M Yadava D, editors. Seed science and technology. Singapore: Springer; 2023. p. 267–98. doi:10.1007/978-981-19-5888-512.

[48] Dadlani M, Yadava DK. Seed science and technology. Singapore: Springer; 2023. p. 440. ISBN 978-981-19-5888-5.

[49] Parsons FG. The early history of seed certification, 1900–1970. In: McDonald M Pardee W, editors. The role of seed certification in the seed industry. CSSA Special Publication Number 10; 1985. p. 3–8. doi:10.2135/cssaspecpub10.c2.

[50] Ajanahalli SR, Tikoo SK, Angadi SP, Kadaru SB, Ajanahalli SR, Vasudeva Rao MJ 2022. Regulatory aspects of the seed business in relation to plant breeding. In: Tiwari A, Tikoo K., Angadi SP, Kadaru SB, Ajanahalli S.R, Vasudeva Rao M.J. (Editors). Market-driven plant breeding for practicing breeders. Springer, Singapore. Pp. 323–87. 10.1007/978-981-19-5434-4_10

[51] OECD. Seed schemes. Promoting the use of certified agriculture seed. 2023 [Accessed 2023 Jan 13]. https://www.oecd.org/agriculture/seeds/#:~:text=The%20OECD%20Schemes%20for%20the,in%20the%2061%20participating%20countries

[52] OECD. OECD schemes for the varietal certification or the control of seed moving in international trade. 2022 [Accessed 2023 Apr 20]. https://www.oecd.org/agriculture/seeds/documents/codes-and-schemes-list-of-varieties-eligible-for-seed-certification.pdf

[53] ESCAA. European Seed Certification Agencies Association. 2023 [Accessed 2023 Jan 23]. http://www.escaa.org/index.php

[54] Akinbo O, Obukosia S, Ouedraogo J, Sinebo W, Savadogo M, Timpo S, Mbabazi R, Maredia K, Makinde D, Ambali A. Commercial release of genetically modified crops in Africa: Interface between biosafety regulatory systems and varietal release systems. Front Plant Sci. 2021;12:1124131. Article. doi:10.3389/fpls.2021.605937. PMC8020716 33828569

[55] Komen J, Wafula DK. Authorizing GM crop varieties: policy implications for seed systems in Sub-Saharan Africa. Agronomy. 2021;11(9):1855. Article. doi:10.3390/agronomy11091855.

[56] Crump DK. Seed certification - an aspect of quality assurance with special reference to white clover seed. In: Hare MD Brock JL editors. Producing herbage seeds. New Zealand Grassland Association Grassland Research and Practice Series No. 2; 1985. p. 75–78.

[57] Jordens R. Progress of plant variety protection based on the international convention for the protection of new varieties of plants (UPOV convention). World Pat Inf. 2005;27(3):232–43. doi:10.1016/j.wpi.2005.03.004.

[58] Srinivasan CS, Shankar B, Holloway GJ. An empirical analysis of the effects of plant variety protection legislation on innovation and transferability. In: Exploring diversity in the european agri -food system. X^th^ European Association of Agricultural Economists (EAAE) International Congress: Zaragoza, Spain; 2002 August 28-31. p. 1–17. doi:10.22004/ag.econ.24788.

[59] Otto HJ. The current status of seed certification in the seed industry. In: McDonald MB Pardee WD, editors. The role of seed certification in the seed industry. CSSA Special Publication Number 10; 1985. p. 9–17. doi:10.2135/cssaspecpub10.c3.

[60] UPOV. UPOV. 2023 [Accessed 2023 Jan 19]. https://www.upov.int/portal/index.html.en

[61] UPOV. Status in relation to UPOV. 2022 [Accessed 2023 Apr 2]. https://www.upov.int/members/en/status_in_relation_to_upov.html

[62] IPONZ. New Zealand intellectual property office. PVR Process. 2023 [Accessed 2023 Apr 2]. https://www.iponz.govt.nz/about-ip/pvr/pvr-process/

[63] Foletto B. European rules for registration of varieties on a national catalogue (and a recommended variety list) for cereals. Proceedings of the COST ACTION 860 – SUSVAR and ECO-PB Workshop on Value for Cultivation and Use testing of organic cereal varieties What are the key issues?; 2008 February 28 and 29; Brussels, Belgium. p. 19–10. http://www.itab.asso.fr/downloads/actes%20suite/proceedings-brussel0208.pdf#page=9

[64] Escajedo San-Epifanio L, Filibi I, Lasa López A, Puigdomènech P, Uncetabarrenechea Larrabe J. Possible EU futures for CRISPR-edited plants: little margin for optimism? Front Plant Sci. 2023;14:1124131. Article. doi:10.3389/fpls.2023.1141455. PMC10061071 37008488

[65] Gov.UK. Add a new plant variety to the national lists. 2022 [Accessed 2023 Jan 30]. https://www.gov.uk/guidance/national-lists-of-agricultural-and-vegetable-crops

[66] Kiewiet B. Plant variety protection in the European community. World Pat Inf. 2005;27(4):319–27. doi:10.1016/j.wpi.2005.07.006.

[67] Van Waes J The VCU variety testing for agricultural crops in an European context. Proceedings of the COST ACTION 860 – SUSVAR and ECO-PB Workshop on Value for Cultivation and Use testing of organic cereal varieties What are the key issues?; 2008 February 28 and 29; Brussels, Belgium. p. 11–15. http://www.itab.asso.fr/downloads/actes%20suite/proceedings-brussel02_08.pdf#page=9

[68] Norwegian Food Safety Authority. Application for listing a plant variety on the Norwegian official list of varieties. 2023 [Accessed 2023 Apr 2]. https://www.mattilsynet.no/planter_og_dyrking/plantesorter/godkjenning/application_for_listing_a_plant_variety_on_the_norwegian_official_list_of_varieties.3298/binary/Application%20for%20listing%20a%20Plant%20Variety%20on%20the%20Norwegian%20Official%20List%20of%20Varieties

[69] Government of Canada. Variety registration. 2020 [Accessed 2023 Apr 3]. https://inspection.canada.ca/plant-varieties/variety-registration/eng/1299175847046/1299175906353

[70] MSP Corporate. State registration of plant varieties. 2023 [Accessed 2023 Apr 2]. https://mspcorporate.com/plant-varieties-registration.htm

[71] Morsy MAM. Variety registration and protection in Egypt. 2009 [Accessed 2023 Apr 7]. https://unece.org/fileadmin/DAM/trade/agr/meetings/ge.06/2009/Egypt_PPTs/S2_MohamedMorsy.pdf

[72] Setimela PS, Badu-Apraku B, Mwangi W. Variety testing and release approaches in DTMA project countries in Sub-Saharan Africa. Harare, Zimbabwe, CIMMYT. 2009 ISBN: 978-92-9059-252-5. https://www.researchgate.net/publication/282604655

[73] Info Trade Kenya. Kenya Gazette supplement No 202. 2016 [Accessed 2023 Apr 2]. https://infotradekenya.go.ke/media/Seeds%20and%20Plant%20Varieties%20Act%20Cap%20326%20-%20Legal%20Notice.pdf

[74] Mwila G. Commentary on the Zambian plant variety and seeds act 1998. In: Halewood M, editor. Farmers’ crop varieties and farmers’ rights challenges in taxonomy and law. Chapter 22. Taylor and Francis, Routledge; 2016. p. 370–72. ISBN:9781136537486. https://books.google.co.nz/books?id=1pWfAQAACAAJ.

[75] Santilli J. Commentary on Brazilian seed law. In: Halewood M, editor. Farmers’ crop varieties and farmers’ rights challenges in taxonomy and law. Chapter 20. Taylor and Francis, Routledge; 2016. p. 338–45. ISBN:9781136537486.https://books.google.co.nz/books?id=1pWfAQAACAAJ.

[76] Medaglia JC. Commentary on the draft proposal for the establishment of a native seed registry in Costa Rica. In: Halewood M, editor. Farmers’ crop varieties and farmers’ rights challenges in taxonomy and law. Chapter 25. Taylor and Francis, Routledge; 2016. p. 385–91. ISBN:9781136537486. https://books.google.co.nz/books?id=1pWfAQAACAAJ.

[77] MOA. Ministry of agriculture, rural development and environment, republic of cyprus. Cultivar Registration Centre. 2023 [Accessed 2023 Jan 30]. http://www.moa.gov.cy/moa/ari/ari.nsf/All/87657456739A0FD9C225786A0023CEA5

[78] Jabatan Pertanian. Varieties registered for national crop list. 2023 [Accessed 2023 Apr 2]. http://pvpbkkt.doa.gov.my/

[79] Blaustein RJ. Commentary in plant variety regulation in the United States of America. In: Halewood M, editor. Farmers’ crop varieties and farmers’ rights challenges in taxonomy and law. Chapter 19. Taylor and Francis, Routledge; 2016. p. 333–37. ISBN:9781136537486. https://books.google.co.nz/books?id=1pWfAQAACAAJ.

[80] PBRA. The National Forage Variety Trials (NFVT) demonstrate how different ryegrass cultivars perform both regionally and nationally. 2023 [Accessed 2023 Jan 30]. https://www.pbra.co.nz/trial-data/forage-grasses/

[81] MLA. Pasture trial network. Meat & Livestock Australia. 2023 [Accessed 2023 Apr 7]. https://www.mla.com.au/extension-training-and-tools/tools-calculators/pasture-trial-network/

[82] GRDC. Groundcover. NVT harvest reports capture latest variety information for WA growers previous article. Grains Research And Development Corporation. 2023 [Accessed 2023 Apr 7]. https://groundcover.grdc.com.au/agronomy/national-variety-trials/nvt-harvest-reports-capture-latest-variety-information-for-wa-growers

[83] Lusk JL, Jamal M, Kurlander L, Roucan M, Taulman L. A meta-analysis of genetically modified food valuation studies. J Agric Resour Econ. 2005;30:28–44.

[84] Ortega DL, Lin W, Ward PS. Consumer acceptance of gene-edited food products in China. Food Qual Prefer. 2022;95:104374. Article. doi:10.1016/j.foodqual.2021.104374.

[85] Cui K, Shoemaker SP 2018. Public perception of genetically-modified (GM) food: a nationwide Chinese consumer study. NPJ Sci Food 2: 10 Article. 10.1038/s41538-018-0018-4 31304260 PMC6550219

[86] Marette S, Disdier A-C, Beghin JC. A comparison of EU and US consumers’ willingness to pay for gene-edited food: evidence from apples. Appetite. 2021;159:105064. Article. doi:10.1016/j.appet.2020.105064. 33278548

[87] Palmieri N, Simeone M, Russo C, Perito MA. Profiling young consumers’ perceptions of GMO products: a case study on Italian undergraduate students. Int J Gastro Food Sci. 2020;21:100224. Article. doi:10.1016/j.ijgfs.2020.100224.

[88] Research First. Public sentiment toward gene editing/advanced breeding solutions. 2022 [Accessed 2023 Jan 17]. https://researchfirst.co.nz/public-perceptions-of-gene-editing/

[89] Ankeny RA, Harms R. Focus groups on consumers’ responses to the use of New Breeding Techniques (NBTs) in food production. The University of Adelaide. 2022 [Accessed 28 Jan 2023]. https://www.foodstandards.gov.au/code/proposals/Documents/FSANZ%20NBT%20final%20report.pdf

[90] Research First. Genetically modified foods. 2023 [Accessed 2023 Mar 18]. https://researchfirst.co.nz/wp-content/uploads/31-Genetically-Modified-Foods-March-2023_v1.1.pdf/

[91] Spendrup S, Eriksson D, Fernqvist F. Swedish consumers´ attitudes and values to genetic modification and conventional plant breeding – the case of fruit and vegetables. GM Crops & Food. 2021;12(1):342–60. doi:10.1080/21645698.2021.1921544. 33970780 PMC8115547

[92] Popek S, Halagarda M. Genetically modified foods: consumer awareness, opinions and attitudes in selected EU countries. Int J Consum Stud. 2017;41(3):325–32. doi:10.1111/ijcs.12345.

[93] Lusk JL, Daniel MS, Mark DR, Lusk CL. Alternative calibration and auction institutions for predicting consumer willingness to pay for nongenetically modified corn chips. J Agric Resour Econ. 2001;26:40–57. https://www.jstor.org/stable/40987094.

[94] Strobbe S, Wesana J, Van Der Straeten D, De Steur H. Public acceptance and stakeholder views of gene edited foods: a global overview. Trends Biotechnol. 2023;41(6):736–40. doi:10.1016/j.tibtech.2022.12.011. 36658005

[95] Devos Y, Ortiz-García S, Hokanson KE, Raybould A. Teosinte and maize × teosinte hybrid plants in Europe−Environmental risk assessment and management implications for genetically modified maize. Agr Ecosyst Environ. 2018;259:19–27. doi:10.1016/j.agee.2018.02.032.

[96] Macall DM, Williams C, Gleim S, Smyth SJ. Canadian consumer opinions regarding food purchase decisions. J Agric Food Res. 2021;3:100098. Article. doi:10.1016/j.jafr.2020.100098.

[97] Shew AM, Nalley LL, Snell HA, Nayga RM, Dixon BL. CRISPR versus GMOs: public acceptance and valuation. Global Food Secur. 2018;19:71–80. doi:10.1016/j.gfs.2018.10.005.

[98] Pappalardo G, D’Amico M, Lusk JL. Comparing the views of the Italian general public and scientists on GMOs. Int J Food Sci Tech. 2021;56(7):3641–50. doi:10.1111/ijfs.14993.

[99] Wunderlich S, Gatto KA. Consumer perception of genetically modified organisms and sources of information. Am Soc Nutr Adv Nutr. 2015;6(6):842–51. doi:10.3945/an.115.008870. PMC4642419 26567205

[100] Morris SH, Adley CC. Genetically modified food issues: attitudes of irish university scientists. Brit Food J. 2000;102(9):669–91. doi:10.1108/00070700010362040.

[101] Ichim MC. The more favorable attitude of the citizens toward GMOs supports a new regulatory framework in the European Union. GM Crops & Food. 2021;12(1):18–24. doi:10.1080/21645698.2020.1795525. 32787504 PMC7553740

[102] Charlebois S, Somogyi S, Music J, Cunningham C. Biotechnology in food: Canadian attitudes towards genetic engineering in both plant-and animal-based foods. BFJ. 2019;121:3181–92. doi:10.1108/BFJ-07-2018-0471.

[103] Smyth SJ. Canadian regulatory perspectives on genome engineered crops. GM Crops & Food. 2017;8(1):35–43. doi:10.1080/21645698.2016.1257468. 27858499 PMC5592975

[104] Borrello M, Cembalo L, Vecchio R, Georgantzis N. Role of information in consumers’ preferences for eco-sustainable genetic improvements in plant breeding. Plos One. 2021;16(7):e0255130. Article. doi:10.1371/journal.pone.0255130. 34324542 PMC8321114

[105] Knight JG, Mather DW, Holdsworth DK, Ermen DF. Acceptance of GM food—an experiment in six countries. Nat Biotechnol. 2007;25(5):507–08. doi:10.1038/nbt0507-507. 17483829

[106] Sendhil R, Yadav S, Prashat PR, Ragupathy R, Rama Prashat PG, Workie E, Ragupathy R, Ramasundaram P. Consumer perception and preference towards genetically modified (GM) foods: bibliometric evidence and policy imperatives. agriRxiv. Wallingford. 2021;2021:42. doi:10.31220/agriRxiv.2021.00061.

[107] Smith MR, König A. Environmental risk assessment for food-related substances. Food Control. 2010;21(12):1588–600. doi:10.1016/j.foodcont.2009.12.032.

[108] Grant WJ, Bray H, Harms R, Ankeny RA, Leach J. Consumer responses to the use of NBTs in the production of food: a systematic literature review. Australian National University. 2021. [Accessed 2023 May 8]. https://www.foodstandards.gov.au/code/proposals/Pages/p1055-definitions-for-gene-technology-and-new-breeding-techniques.aspx

[109] FSANZ. Final report. Review of food derived using new breeding techniques. 2019 [Accessed 5 Jun 2023]. https://www.foodstandards.gov.au/consumer/gmfood/Documents/NBT%20Final%20report.pdf

[110] Reader’s Digest. Misinformation vs. Disinformation: how to tell the difference. 2023 [Accessed 2023 Jun 10]. https://www.rd.com/article/misinformation-vs-disinformation/

[111] Lynas M, Adams J, Conrow J. Misinformation in the media: global coverage of GMOs 2019-2021. GM Crops & Food; 2022. doi:10.1080/21645698.2022.2140568. PMC11702960 36384421

[112] Lassoued R, Macall DM, Smyth SJ, Phillips PW, Hesseln H. How should we regulate products of new breeding techniques? Opinion of surveyed experts in plant biotechnology. Biotechnol Reports. 2020;e00460. doi:10.1016/j.btre.2020.e00460. PMC7322807 32617264

[113] Ventura L. 5 influential activist NGOs spreading crop biotechnology misinformation in Latin America. 2023 [Accessed 2023 Jun 5]. https://geneticliteracyproject.org/2023/05/19/top-5-activist-groups-spreading-anti-gmo-myths-in-latin-america/

[114] Roberts P. Kenyans subjected to world’s worst misinformation on GMOs, study finds. Alliance For Science. 2023 [Accessed 2023 Jun 5]. https://allianceforscience.org/blog/2023/02/kenyans-subjected-to-worlds-worst-misinformation-on-gmos-study-finds/#:~:text=An%20earlier%20Alliance%20for%20Science,makes%20it%20the%20world’s%20worst

[115] GE Free NZ 2023. [Accessed 2023 Jun 5]. https://www.gefree.org.nz/

[116] Fernbach PM, Light N, Scott SE, Inbar Y, Rozin P. Extreme opponents of genetically modified foods know the least but think they know the most. Nat Hum Behav. 2019;3:251–56. doi:10.1038/s41562-018-0520-3. 30953007

[117] OECD. Safety evaluation of foods derived by modern biotechnology. Paris, France, OECD. 1993 [Accessed 2023 Apr 20]. http://www.oecd.org/dataoecd/37/18/41036698.pdf

[118] OECD. Test No. 408: repeated dose 90-day oral toxicity study in rodents. In: OECD guidelines for the testing of chemicals, section, 4, health effects. Paris, France: OECD; 1998 [Accessed 2023 Apr 20]. https://www.oecd-ilibrary.org/docserver/9789264070707-en.pdf?expires=1654508260&id=id&accname=guest&checksum=05CFAC4B1B5D0517CAF3B24EDFCCC20F.

[119] Pusztai A. Genetically modified foods: Are they a risk to human/animal health? Action Bioscience. 2001 [Accessed 2023 Apr 20] https://www.globalmagazine.info/sites/default/files/PDF/pusztai-gm-foods-risk-human-animal-health-2001.pdf

[120] Pusztai A. Can science give us the tools for recognizing possible health risk of GM? Nutr Health. 2002;16:73–84. doi:10.1177/026010600201600202. 12102369

[121] Smyth SJ, Phillips PWB. Risk, regulation and biotechnology: the case of GM crops. GM Crops & Food. 2014;5(3):170–77. doi:10.4161/21645698.2014.945880. 25437235 PMC5033226

[122] Craig W, Tepfer M, Degrassi G, Ripandelli D. An overview of general features of risk assessments of genetically modified crops. Euphytica. 2008;164(3):853–80. doi:10.1007/s10681-007-9643-8.

[123] EFSA GMO Panel Working Group on Animal Feeding Trials. Safety and nutritional assessment of GM plants and derived food and feed: the role of animal feeding trials. Food Chem Toxicol. 2008;46(Suppl 1):S2–70. doi:10.1016/j.fct.2008.02.008. 18328408

[124] Gizaw Z. Public health risks related to food safety issues in the food market: a systematic literature review. Environ Health Prev Med. 2019;24(1):68. Article. doi:10.1186/s12199-019-0825-5. 31785611 PMC6885314

[125] Hull RO, Bosse MA, Tzotzos GE. Training for implementing risk assessment regulations for the release of GM crops. Aspects Appl Biol. 2009;96:1–8.

[126] van den Belt H. Debating the precautionary principle: “guilty until proven innocent” or “Innocent until proven guilty”? Plant Physiol. 2003;132(3):1122–26. doi:10.1104/pp.103.023531. 12857792 PMC526264

[127] Burke D. GM food and crops: what went wrong in the UK? Many of the public’s concerns have little to do with science. EMBO Rep. 2004;5(5):432–36. doi:10.1038/sj.embor.7400160. 15184970 PMC1299063

[128] Belderok A, de Vries AG, van der Spek L. Genetically modified organisms in food and beverages. Roland Berger Report, The Netherland. 2021 [Accessed 5 Jun 2023]. https://www.rolandberger.com/en/Insights/Publications/Genetically-modified-organisms-in-food-and-beverages.html

[129] DeFrancesco L. How safe does transgenic food need to be? Nat Biotechnol. 2013;31(9):794–802. doi:10.1038/nbt.2686. 24022153

[130] Dunn SE, Vicini JL, Glenn KC, Fleischer DM, Greenhawt MJ. The allergenicity of genetically modified foods from genetically engineered crops: a narrative and systematic review. Ann Allergy, Asthma Immunol. 2017;119:214–22. doi:10.1016/j.anai.2017.07.010. 28890018

[131] Ladics GS. Assessment of the potential allergenicity of genetically-engineered food crops. J Immunotoxicol. 2019;16(1):43–53. doi:10.1080/1547691X.2018.1533904. 30409058

[132] Nicolia A, Manzo A, Veronesi F, Rosellini D. An overview of the last 10 years of genetically engineered crop safety research. Crit Rev Biotechnol. 2014;34(1):77–88. doi:10.3109/07388551.2013.823595. 24041244

[133] Panchin AY, Tuzhikov AI. Published GMO studies find no evidence of harm when corrected for multiple comparisons. Crit Rev Biotechnol. 2017;37(2):213–17. doi:10.3109/07388551.2015.1130684. 26767435

[134] Sasson A. Genetically modified crops (GM crops) and derived foods: brief review of their impact on health and environment, and of their social acceptance. Front In Sci Eng. 2018 [Accessed 20 Apr 2023];8:1–52. https://revues.imist.ma/index.php/fsejournal/article/download/27990/14625.

[135] The Royal Society. Is it safe to eat GM crops? 2016 [Accessed 2023 Jan 1]. https://royalsociety.org/topics-policy/projects/gm-plants/is-it-safe-to-eat-gm-crops/

[136] Bernstein JA, Bernstein IL, Bucchini L, Goldman LR, Hamilton RG, Lehrer S, Rubin C, Sampson HA. Clinical and laboratory investigation of allergy to genetically modified foods. Environ Health Perspectives. 2003;111(8):1114–21. doi:10.1289/ehp.5811. PMC1241560 12826483

[137] McHughen A. The regulation of GM foods: who represents the public interest? Int J. 2000;55(4):624–32. doi:10.1177/002070200005500406.

[138] Caradus JR. Intended and unintended consequences of genetically modified crops – myth, fact and/or manageable outcomes? New Zeal J Agr Res. 2022;65:1–101. doi:10.1080/00288233.2022.2141273.

[139] Ladics GS, Bartholomaeus A, Bregitzer P, Doerrer NG, Gray A, Holzhauser T, Jordan M, Keese P, Kok E, Macdonald P, et al. Genetic basis and detection of unintended effects in genetically modified crop plants. Transgenic Res. 2015;24(4):587–603. doi:10.1007/s11248-015-9867-7. 25716164 PMC4504983

[140] Lack G. Clinical risk assessment of GM foods. Toxicol Lett. 2002;127:337–40. doi:10.1016/S0378-4274(01)00517-3. 12052675

[141] Nordlee JA, Taylor SL, Townsend JA, Thomas LA, Bush RK. Identification of a Brazil-nut allergen in transgenic soybeans. N Engl J Med. 1996;334(11):688–92. doi:10.1056/NEJM199603143341103. 8594427

[142] van Baalen S, Srinivas KR, He G. Challenges of global technology assessment in biotechnology—bringing clarity and better understanding in fragmented global governance. In: Hennen L, Hahn J, Ladikas M, Lindner R, Peissl W van Est R., editors. Technology assessment in a globalized world: facing the challenges of transnational technology governance. Cham:Springer International Publishing; 2023. p. 149–73. doi:10.1007/978-3-031-10617-0_8.

[143] FAO/WHO. Consultation on the assessment of biotechnology in food production and processing as related to food safety (‎1990: Geneva, Switzerland)‎. Strategies for assessing the safety of foods produced by biotechnology: report of a joint FAO/WHO consultation [‎held in Geneva from 5 to 10 November 1990]. World Health Organization. 1991. https://apps.who.int/iris/handle/10665/41465

[144] United Nations Treaty Collection. Chapter XXVII Environment 8. A Cartagena Protocol on Biosafety to the Convention on Biological Diversity, Montreal, 29 January 2000. 2023 [Accessed 13 Jan 2023]. https://treaties.un.org/Pages/ViewDetails.aspx?src=IND&mtdsg_no=XXVII-8-a&chapter=27&clang=_en#top

[145] ISAAA. Pocket K No. 8: Cartagena Protocol on Biosafety. 2004 [Accessed 2023 Jan 13]. https://www.isaaa.org/resources/publications/pocketk/8/default.asp

[146] Convention on Biological Diversity. The cartagena protocol on biosafety. 2023 [Accessed 2023 Jan 13]. https://bch.cbd.int/protocol/text/

[147] Smyth SJ. Genetically modified crops, regulatory delays, and international trade. Food Energy Secur. 2017;6:78–86. doi:10.1002/fes3.100.

[148] Araki M, Ishii T. Towards social acceptance of plant breeding by genome editing. Trends Plant Sci. 2015;20(3):145–49. doi:10.1016/j.tplants.2015.01.010. 25726138

[149] Convention on Biological Diversity. Text of the Cartagena Protocol on Biosafety. 2021 [Accessed 2023 May 11]. https://bch.cbd.int/protocol/text/

[150] Kerr WA. Worlds apart on GMOs—Can trade agreements bridge the gap? NABC report 27. stewardship for the sustainability of genetically engineered crops: the way forward in pest management, coexistence, and trade. 2015. p. 133–45. https://hdl.handle.net/1813/51463.

[151] Holtby KL, Kerr WA, Hobbs JE. International environmental liability and barriers to trade. Cheltenham UK: Edward Elgar; 2007. p. 192. ISBN: 978184720 0976. https://ideas.repec.org/b/elg/eebook/12610.html.

[152] Hobbs AL, Hobbs JE, Kerr WA. The biosafety protocol: Multilateral agreement on protecting the environment or protectionist club? J World Trade. 2005;39:281–300. doi:10.54648/TRAD2005024.

[153] FAO/WHO. Codex Alimentarius, international food standards. Biotechnology. 2023 [Accessed 2023 Jan 31]. https://www.fao.org/fao-who-codexalimentarius/thematic-areas/biotechnology/en/

[154] FAO. Global community meeting of the FAO GM Foods Platform. Towards effective risk-based GM food safety assessment and regulatory management. Meeting Report. Rome; 2020. doi:10.4060/ca8945en

[155] Vega Rodríguez A, Rodríguez-Oramas C, Sanjuán Velázquez E, Hardisson de la Torre A, Rubio Armendáriz C, Carrascosa Iruzubieta C. Myths and realities about genetically modified food: a risk-benefit analysis. Appl Sci. 2022;12(6):2861. Article. doi:10.3390/app12062861.

[156] FAO. Guideline for the conduct of food safety assessment of foods derived from recombinant-DNA plants. CAC/GL 45-2003. 2003 [Accessed 2023 Jan 17]. https://www.fao.org/fileadmin/user_upload/gmfp/docs/CAC.GL_45_2003.pdf

[157] Tsakok I, Mengoub FE 2021. Genetically modified organisms: promising or problematic for food security? A review of major developments in selected industrialized countries Part I. Policy Brief, Policy Centre For The New South. https://www.policycenter.ma/sites/default/files/PB_21-02_Tsakok-Mengoub.pdf

[158] Genetic Literacy Project. Human and Agriculture Gene Editing: Regulations and Index. 2023 [Accessed 2023 Jan 28]. https://crispr-gene-editing-regs-tracker.geneticliteracyproject.org/

[159] Ishii T, Araki M. A future scenario of the global regulatory landscape regarding genome edited crops. GM Crops & Food; 2017. doi:10.1080/21645698.2016.1261787. PMC5592978 27960622

[160] Royal Society Te Apārangi. Gene editing scenarios in the primary industries. Wellington, New Zealand: Royal society Te Apārangi; 2019. p. 36. ISBN: 978-1-877264-41-2.

[161] OECD. Developments in delegations on the safety assessment of novel foods and feeds, June 2022 – April 2023. Series On The Safety Of Novel Foods And Feeds No. 36. 2023 [Accessed 2023 Aug 5].https://one.oecd.org/document/ENV/CBC/MONO(2023)29/en/pdf

[162] OECD. Biotechnology update - Internal Co-ordination Group for Biotechnology (ICGB) No. 41 – June 2022. Meeting of the Environment Policy Committee (EPOC) at ministerial level. 2022 [Accessed 2023 Jan 17]. https://www.oecd.org/chemicalsafety/biotrack/biotech-update-issue-41-june-2022.pdf

[163] Friedrichs S, Takasu Y, Kearns P, Dagallier B, Oshima R, Schofield J, Moreddu C. Policy considerations regarding genome editing. Trends Biotechnol. 2019;37(10):1029–32. doi:10.1016/j.tibtech.2019.05.005. 31229272

[164] WTO. Uruguay round agreement: trips part II — standards concerning the availability, scope and use of intellectual property rights, sections 5 and 6. 2023 [Accessed 2023 Apr 17]. https://www.wto.org/english/docs_e/legal_e/27-trips_04c_e.htm

[165] McAfee K. Neoliberalism on the molecular scale. Economic and genetic reductionism in the biotechnology battles. Geoforum. 2003;34:203–19. doi:10.1016/S0016-7185(02)00089-1.

[166] USDA. WTO members support policy approaches to enable innovation in agriculture. 2018 [Accessed 2023 Jan 30]. https://www.usda.gov/media/press-releases/2018/11/02/wto-members-support-policy-approaches-enable-innovation-agriculture

[167] WTO. International statement on agricultural applications of precision biotechnology. 2018 [Accessed 2023 Jan 30]. https://docs.wto.org/dol2fe/Pages/FE_Search/FE_S_S009-DP.aspx?language=E&CatalogueIdList=250406,249838,249823,249748,249641,249507,249371,249321,249324,249267&CurrentCatalogueIdIndex=7&FullTextHash=&HasEnglishRecord=True&HasFrenchRecord=True&HasSpanishRecord=True

[168] Hobbs JE, Kerr WA, Smyth SJ. The perils of zero tolerance: Technology management, supply chains and thwarted globalization. IJTG. 2014;7:203–16. doi:10.1504/IJTG.2014.064742.

[169] Isaac GE. Sanitary and phytosanitary issues. In: In: Kerr WA Gaisford JD., editors. Handbook on international trade policy. Cheltenham UK:Edward Elgar Publishing; 2007. pp. 383–93. doi:10.4337/9781847205469.00047.

[170] Koop A. Top heavy: Countries by share of the global economy. 2022 [Accessed 2023 Jan 29]. https://www.visualcapitalist.com/countries-by-share-of-global-economy/

[171] World Population Review. GDP Ranked by Country 2023. 2023 [Accessed 2023 Jan 29]. https://worldpopulationreview.com/countries/by-gdp

[172] WorldData. Biggest economies in 2021 by gross domestic product. 2021. https://www.worlddata.info/largest-economies.php

[173] Worldometer. GDP by country. 2017 [Accessed 2023 Jan 29]. https://www.worldometers.info/gdp/gdp-by-country/

[174] ISAAA. GM approval database. Ithaca: International Service for the Acquisition of Agri-biotech Applications ISAAA. 2023 [Accessed 2023 Jun 20]. https://www.isaaa.org/gmapprovaldatabase/default.asp

[175] Nang’ayo F, Simiyu-Wafukho S, Oikeh SO. Regulatory challenges for GM crops in developing economies: the African experience. Transgenic Res. 2014;23(6):1049–55. doi:10.1007/s11248-014-9805-0. 24821674

[176] Schnurr MA, Gore C. Getting to ‘Yes’: governing genetically modified crops in Uganda. J Int Dev. 2015;27(1):55–72. doi:10.1002/jid.3027.

[177] Mousa H. Agricultural biotechnology annual - Saudi Arabia. Biotechnology and other new production technologies. USDA Foreign Agricultural Service. 2022 [Accessed 2023 Jan 29]. https://apps.fas.usda.gov/newgainapi/api/Report/DownloadReportByFileName?fileName=Agricultural%20Biotechnology%20Annual_Riyadh_Saudi%20Arabia_SA2022-0015.pdf

[178] Entine J, Felipe MSS, Groenewald JH, Kershen DL, Lema M, McHughen A, Nepomuceno AL, Ohsawa R, Ordonio RL, Parrott WA, et al. Regulatory approaches for genome edited agricultural plants in select countries and jurisdictions around the world. Transgenic Res. 2021;30(4):551–84. doi:10.1007/s11248-021-00257-8. 33970411 PMC8316157

[179] National Academies of Sciences, Engineering, and Medicine. Genetically engineered crops: experiences and prospects. Washington, DC: The National Academies Press; 2016. p. 607. doi:10.17226/23395. 28230933

[180] Turnbull C, Lillemo M, Hvoslef-Eide TAK. Global regulation of genetically modified crops amid the gene edited crop boom – a review. Front Plant Sci. 2021;12:630396. Article. doi:10.3389/fpls.2021.630396. 33719302 PMC7943453

[181] Regulations. Pesticides: exemptions of certain plant-incorporated protectants derived from newer technologies. posted by the environmental protection agency on May 31, 2023. 2023 [Accessed 2023 Jun 8]. https://www.regulations.gov/document/EPA-HQ-OPP-2019-0508-0122

[182] Stokstad E. EPA decision to tighten oversight of gene-edited crops draws mixed response. Scienceinsider. 2023 [Accessed 2023 Jun 8]. https://www.science.org/content/article/epa-decision-tighten-oversight-gene-edited-crops-draws-mixed-response

[183] EPA. Modernizing the regulatory system for biotechnology products: final version of the 2017 update to the coordinated framework for the regulation of biotechnology. Washington, DC: EPA. 2017 [Accessed 2023 Jan 29]. https://www.epa.gov/regulation-biotechnology-under-tsca-and-fifra/modernizing-regulatory-system-biotechnology-products

[184] The White House. Increasing the transparency, coordination, and predictability of the biotechnology regulatory system. 2017 [Accessed 2023 Jan 29]. https://obamawhitehouse.archives.gov/blog/2017/01/04/increasing-transparency-coordination-and-predictability-biotechnology-regulatory

[185] Wolt JD, Wolf C. Policy and governance perspectives for regulation of genome edited crops in the United States. Front Plant Sci. 2018;9:1606. Article. doi:10.3389/fpls.2018.01606. 30467510 PMC6236124

[186] Xiao Z, Kerr WA. Biotechnology in China – regulation, investment, and delayed commercialization. GM Crops & Food. 2022;13(1):86–96. doi:10.1080/21645698.2022.2068336. 35506348 PMC9090284

[187] Postings R. China approves first gene edited crop in food security push. 2023 [Accessed 2023 Aug 5]. https://impakter.com/china-approves-first-gene-edited-crop-in-food-security-push/#:~:text=China%20has%20approved%20the%20safety,owned%20Shandong%20Shunfeng%20Biotechnology%20Company.

[188] Patton D. China plans overhaul of seed rules to pave way for GMO approvals. Reuters. 2021 [Accessed 2023 Aug 5]. https://www.reuters.com/article/china-gmo-regulations-idINKBN2HZ0D3

[189] Patton D. China to allow gene-edited crops in push for food security. Reuters. 2022 [Accessed 2023 Aug 5]. https://www.reuters.com/world/china/china-drafts-new-rules-allow-gene-edited-crops-2022-01-25/

[190] FFTC. The Japanese policy for genetically modified food. 2017 [Accessed 2023 Jan 29]. https://ap.fftc.org.tw/article/1169

[191] Ministry of the Environment. Japan biosafety clearing house (J-BCH). Domestic law and regulations. 2017 [Accessed 2023 Jan 29]. https://www.biodic.go.jp/bch/english/e_index.html

[192] Ishii T. Regulation of genome editing in plant biotechnology: japan. In: Dederer, HG. Hamburger, D., editors. Regulation of genome editing in plant biotechnology. Cham: Springer International Publishing; 2019. p. 239–62. doi:10.1007/978-3-030-17119-3_6.

[193] Tsuda M, Watanabe KN, Ohsawa R. Regulatory status of genome-edited organisms under the Japanese cartagena act. Frontiers In Bioeng Biotechnol. 2019;7:387. Article. doi:10.3389/fbioe.2019.00387. PMC6908812 31867318

[194] Lemke SL. Gene editing in plants. a nutrition professional’s guide to the science, regulatory, and social considerations. Nutr Commun. 2022;57:57–63. doi:10.1097/NT.0000000000000532.

[195] Sanatechseed 2021. Launch of genome edited tomato fruit for purchase. [Accessed 2023 Jan 29]. https://sanatech-seed.com/en/20210915-2/

[196] WELL_NZ. Modern genetic technology – what it is and how it is regulated. A reference document from Te Puna Whakaaronui: New Zealand’s independent food and fibre sector think tank; 2023. ISBN: 978-1-99-106276-5.

[197] Brookes G. Twenty-one years of using insect resistant (GM) maize in Spain and Portugal: farm-level economic and environmental contributions. GM Crops & Food. 2019;10(2):90–101. doi:10.1080/21645698.2019.1614393. 31072184 PMC6615534

[198] Isaac GE, Kerr WA. Whose vision of the future? The entrenched international conflict over genetic modification. Geneva Post Q. 2007;2:87–107.

[199] Viju C, Yeung MT. The trade implications of the post-moratorium European Union approval system for genetically modified organisms. J World Trade. 2012;46:1207–38. doi:10.54648/TRAD2012037.

[200] Tagliabue G. The meaningless pseudo‐category of “GMOs”. The trouble with the “new techniques” for genetically modifying crops demonstrates the illogical process‐based definition of GMOs in EU regulation. EMBO Rep. 2016;17:10–13. doi:10.15252/embr.201541385. 26559524 PMC4718412

[201] Smyth SJ. Regulatory barriers to improving global food security. Global Food Secur. 2020;26:100440. Article. doi:10.1016/j.gfs.2020.100440. PMC7521901 33014703

[202] Sandin P, Munthe C, Björnberg KE. Technology neutrality in European regulation of GMOs. Ethics, Policy & Environ. 2022;25(1):52–68. doi:10.1080/21550085.2020.1865085.

[203] Eriksson D. The Swedish policy approach to directed mutagenesis in a European context. Physiol Plant. 2018;164(4):385–95. doi:10.1111/ppl.12740. 29602252

[204] Official Journal of the European Communities 2001. Directive 2001/18/EC of the European parliament and of the council of 12 March 2001 on the deliberate release into the environment of genetically modified organisms and repealing council directive 90/220/EEC. L 106/1. Off J Euro Commun. https://eur-lex.europa.eu/LexUriServ/LexUriServ.do?uri=CONSLEG:2001L0018:20080321:EN:PDF

[205] Eckerstorfer MF, Dolezel M, Engelhard M, Giovannelli V, Grabowski M, Heissenberger A, Lener M, Reichenbecher W, Simon S, Staiano G, et al. Recommendations for the assessment of potential environmental effects of genome editing applications in plants in the EU. Plants. 2023;12:1764. Article. doi:10.3390/plants12091764. 37176822 PMC10180588

[206] Guehlstorf NP, Hallstrom LK. The role of culture in risk regulations: a comparative case study of genetically modified corn in the United States of America and European Union. Environ Science & Policy. 2005;8(4):327–42. doi:10.1016/j.envsci.2005.04.007.

[207] Anyshchenko A, Yarnold J. From ‘mad cow’ crisis to synthetic biology: challenges to EU regulation of GMOs beyond the European context. Int Environ Agreements: Polit, Law Econ. 2021;21:391–404. doi:10.1007/s10784-020-09516-1.

[208] De Marchi B, Ravet JR. Risk management and governance: a post-normal science approach. Sci Direct. 1999;31:743–57. doi:10.1016/S0016-3287(99)00030-0.

[209] Davison J. GM plants: science, politics and EC regulations. Plant Sci. 2010;178(2):94–98. doi:10.1016/j.plantsci.2009.12.005.

[210] Henseler M, Piot-Lepetit I, Ferrari E, Mellado AG, Banse M, Grethe H, Parisi C, Hélaine S. On the asynchronous approvals of GM crops: potential market impacts of a trade disruption of EU soy imports. Food Policy. 2013;41:166–76. doi:10.1016/j.foodpol.2013.05.005.

[211] Sieradzki Z, Mazur M, Król B, Kwiatek K. Prevalence of genetically modified soybean in animal feeding stuffs in poland. J Veterinary Res. 2021;65(1):93–99. doi:10.2478/jvetres-2021-0012. PMC8009591 33817401

[212] Turkec A, Lucas SJ, Karlik E. Monitoring the prevalence of genetically modified (GM) soybean in Turkish food and feed products. Food Control. 2016;59:766–72. doi:10.1016/j.foodcont.2015.06.052.

[213] ISAAA. GM approval database – commercial GM traits list. GM crop events approved in European Union. International Service For The Acquisition Of Agri-Biotech Applications. Ithaca, NY. 2022 [Accessed 2023 May 11]. https://www.isaaa.org/gmapprovaldatabase/approvedeventsin/default.asp?CountryID=EU&Country=European%20Union

[214] European Commission. GMO authorisation. 2023 [Accessed 2023 Aug 5]. https://food.ec.europa.eu/plants/genetically-modified-organisms/gmo-authorisation_en

[215] EUR-Lex. Document 52023PC0411. Proposal for a REGULATION of the EUROPEAN PARLIAMENT and of the COUNCIL on plants obtained by certain new genomic techniques and their food and feed, and amending Regulation (EU) 2017/625. COM/2023/411 final. 2023 [Accessed 2023 Aug 5]. https://eur-lex.europa.eu/legal-content/EN/ALL/?uri=COM:2023:411:FIN

[216] Roger CR. NGT: the European Commission plays a” simultaneously” approach. European Scientist. 2023 [Accessed 2023 Aug 6]. https://www.europeanscientist.com/en/features/ngt-the-european-commission-plays-a-simultaneously-approach/#

[217] European Commission. Proposal for a REGULATION of the EUROPEAN PARLIAMENT and of the COUNCIL on plants obtained by certain new genomic techniques and their food and feed, and amending Regulation (EU) 2017/625. 2023 [Accessed 2023 Aug 5]. https://eur-lex.europa.eu/resource.html?uri=cellar:c88fe9ac-1c06-11ee-806b-01aa75ed71a1.0001.02/DOC_1&format=PDF

[218] Verma V, Negi S, Kumar P, Srivastava DK. Global status of genetically modified crops. In: Kumar Srivastava, D., Kumar Thakur, A. Kumar, P., editors. Agricultural biotechnology: latest research and trends. Singapore: Springer Nature Singapore; 2021. p. 305–22. doi:10.1007/978-981-16-2339-4_13.

[219] Chimata MK, Bharti G. Regulation of genome edited technologies in India. Transgenic Res. 2019;28(S2):175–81. doi:10.1007/s11248-019-00148-z. 31321702

[220] GEAC. Genetic engineering appraisal committee, ministry of environment, forest and climate change, government of India. 2017 [Accessed 2023 Jun 10]. http://geacindia.gov.in/

[221] Ministry of Science and Technology, Government of India. Draft document on genome edited organisms: regulatory framework and guidelines for risk assessment. 2020 [Accessed 2023 Feb 20]. https://dbtindia.gov.in/sites/default/files/DraftRegulatoryFrameworkGenomeEditing9jan2020a.pdf

[222] Bhattacharya A, Parkhi V, Char B. Genome editing for crop improvement: a perspective from India. In Vitro Cell Dev Biol -Plant. 2021;57:565–73. doi:10.1007/s11627-021-10184-2. 34075289 PMC8152710

[223] Barse B, Yazdani SS. India—GMOs/Synthetic biology rules/regulations and biodiversity – a legal perspective from India. In: Chaurasia, A., Hawksworth, D.L. Pessoa de Miranda, M., editors. Gmos. topics in biodiversity, conservation and ecological processes. Cham: Springer; 2020. p. 559–76. doi:10.1007/978-3-030-53183-6_32.

[224] Parallel Parliament. Genetic technology (precision breeding) act 2023. 2023 [Accessed 2023 Aug 5]. https://www.parallelparliament.co.uk/bills/2022-23/genetictechnologyprecisionbreeding#:~:text=make%20provision%20about%20the%20release,animals%3B%20and%20for%20connected%20purposes.

[225] Legislation.gov.uk. Genetic Technology (Precision Breeding) Act 2023. 2023 [Accessed 2023 Aug 5]. https://www.legislation.gov.uk/ukpga/2023/6/contents/enacted

[226] Smyth S, McHughen A. Regulating innovative crop technologies in Canada: the case of regulating genetically modified crops. Plant Biotechnol J. 2008;6(3):213–25. doi:10.1111/j.1467-7652.2007.00309.x. 18028290

[227] Gupta S, Kumar A, Patel R, Kumar V. Genetically modified crop regulations: scope and opportunity using the CRISPR-Cas9 genome editing approach. Mol Biol Rep. 2021;48(5):4851–63. doi:10.1007/s11033-021-06477-9. 34114124

[228] Buchholzer M, Frommer WB. An increasing number of countries regulate genome editing in crops. New Phytol. 2022;237:12–15. doi:10.1111/nph.18333. 35739630

[229] ASSAf. Academy of science of South Africa. Regulation of Agricultural GM Technology in Africa. 2012 [Accessed 2023 Jan 28].https://research.assaf.org.za/bitstream/handle/20.500.11911/81/2012_assaf_K9610ASSAFGMOReportDevV8LR.pdf?sequence=1

[230] Quemada H. Lessons learned from the introduction of genetically engineered crops: relevance to gene drive deployment in Africa. Transgenic Res. 2022;31(3):285–311. doi:10.1007/s11248-022-00300-2. 35545692 PMC9135826

[231] Paarlberg R. Starved for science: how biotechnology is being kept out of Africa. Cambridge, MA and London, England: Harvard University Press; 2009. p. 93. ISBN-13:978-0-674-02973-6.

[232] Adenle AA, Morris EJ, Parayil G. Status of development, regulation and adoption of GM agriculture in Africa: views and positions of stakeholder groups. Food Policy. 2013;43:159–66. doi:10.1016/j.foodpol.2013.09.006.

[233] Zetterberg C, Björnberg EK. Time for a new EU regulatory framework for GM crops? J Agric Environ Ethics. 2017;30:325–47. doi:10.1007/s10806-017-9664-9.

[234] Morris S, Spillane C. EU GM crop regulation: a road to resolution or a regulatory roundabout? Eur J Risk Regul. 2010;1:359–69. doi:10.1017/S1867299X00000805.

[235] Sommer A, West KP JrEditors. Vitamin a deficiency: health, survival and vision. Oxford University Press: New York; 1996. pp. 1–438. ISBN 0-19-508824-7.

[236] Beyer P. Golden Rice and ‘Golden’ crops for human nutrition. N Biotechnol. 2010;27(5):478–81. doi:10.1016/j.nbt.2010.05.010. 20478420

[237] Potrykus I. Lessons from the ‘humanitarian golden rice’ project: regulation prevents development of public good genetically engineered crop products. N Biotechnol. 2010;27(5):466–72. doi:10.1016/j.nbt.2010.07.012. 20650337

[238] Wesseler J, Zilberman D. The economic power of the golden rice opposition. Environ Dev Econ. 2014;19:724–42. doi:10.1017/S1355770X1300065X.

[239] Wu F, Wesseler J, Zilberman D, Russell RM, Chen C, Dubock AC. Allow golden rice to save lives. Proc Natl Acad Sci USA. 2021;118:e2120901118. doi:10.1073/pnas.212090111. 34911769 PMC8713968

[240] Ye X, Al-Bbili S, Kloti A, Zhang J, Lucca P, Beyer P, Potrykus I. Engineering the provitamin a (β-Carotene) biosynthetic pathway into (carotenoid-free) rice endosperm. Sci. 2000;287:303–05. doi:10.1126/science.287.5451.303. 10634784

[241] Ensering M. Tough lessons from golden rice. Sci. 2008;320:468–71. doi:10.1126/science.320.5875.468. 18436769

[242] Tang G, Qin J, Dolnikowski GG, Russell RM, Grusak MA. Golden rice is an effective source of vitamin a. Am J Clin Nutr. 2009;89(6):1776–83. doi:10.3945/ajcn.2008.27119. 19369372 PMC2682994

[243] Sumangil FR. 7 provinces to produce ‘golden rice’. The Manila Times. 2022, May 27 [Accessed 2022 June 22]. https://www.manilatimes.net/2022/05/27/news/regions/7-provinces-to-produce-golden-rice/1845154

[244] Rosell E. Stop opposing healthy food for poor children, Greenpeace. Warp News. 2023 [Accessed 2023 Jun 6]. https://www.warpnews.org/column/stop-opposing-healthy-food-for-poor-children-greenpeace/

[245] Anderson K, Jackson LA, Nielsen CP. GM rice adoption: impact for welfare and poverty alleviation. J Eco Inte. 2005;20:125–34. doi:10.11130/jei.2005.20.4.771.

[246] Stein AJ, Sachdev HPS, Qaim M. Potential impact and cost effectiveness of golden rice. Nat Biotechnol. 2006;24:1200–01. doi:10.1038/nbt1006-1200b. 17033649

[247] Staver C, Pemsl DE, Scheerer L, Perez Vicente L, Dita M. Ex ante assessment of returns on research investments to address the impact of Fusarium wilt tropical race 4 on global banana production. Front Plant Sci. 2020;11:844. Article. doi:10.3389/fpls.2020.00844. 32733497 PMC7357546

[248] de Figueiredo Silva F, Kaplan S, Tobar FAM, Potts MD, Martinez RLE, Zilberman D. Estimating worldwide benefits from improved bananas resistant to fusarium wilt tropical race 4. J Of Agr App Econ Assoc. 2023;2:20–34. doi:10.1002/jaa2.41.

[249] Ainembabazi JH, Tripathi L, Rusike J, Abdoulaye T, Manyong V, Lee S-W. Ex-ante economic impact assessment of genetically modified banana resistant to *Xanthomonas* wilt in the Great Lakes Region of Africa. PLoS One. 2015;10(9):e0138998. Article. doi:10.1371/journal.pone.0138998. 26414379 PMC4587572

[250] Nawaz MA, Ali MA, Golokhvast KS, Tsatsakis AM, Chung G. Gmos: history, economic status, risks, and socio-economic regulatory frameworks. In: Nawaz MA, Chung G, Golokhvast KS Tsatsakis A., editors. Gmos and political stance. Academic Press; 2023. p. 1–13. 10.1016/B978-0-12-823903-2.00013-5

[251] Brookes G, Barfoot P. GM crops: global socio-economic and environmental impacts 1996-2018. 2020 [Accessed 2022 Jun 9]. https://www.pgeconomics.co.uk/pdf/globalimpactfinalreportJuly2020.pdf

[252] Brookes G. Farm income and production impacts from the use of genetically modified (GM) crop technology 1996-2020. GM Crops & Food. 2022;13(1):171–95. doi:10.1080/21645698.2022.2105626. 35983931 PMC9397136

[253] Brookes G, Barfoot P. GM crop technology use 1996-2018: farm income and production impacts. GM Crops & Food. 2020;11(4):242–61. doi:10.1080/21645698.2020.1779574. 32706314 PMC7518751

[254] Scheitrum D, Schaefer KA, Nes K. Realized and potential global production effects from genetic engineering. Food Policy. 2020;93:101882. Article. doi:10.1016/j.foodpol.2020.101882.

[255] Schiek B, Hareau G, Baguma Y, Medakker A, Douches D, Shotkoski F, Ghislain M. Demystification of GM crop costs: releasing late blight resistant potato varieties as public goods in developing countries. Int J Biotechnol. 2016;14:112–31. doi:10.1504/IJBT.2016.077942.

[256] McDougall P. The cost and time involved in the discovery, development, and authorization of a new plant biotechnology derived trait, a consultancy study for crop life international. Midlothian, UK. 2011 [Accessed 2023 Apr 8]. http://www.croplife.org/PhillipsMcDougallStudy

[257] Kalaitzandonakes N, Alston JM, Bradford KJ. Compliance costs for regulatory approval of new biotech crops. Nat Biotechnol. 2007;25(5):509–11. doi:10.1038/nbt0507-509. 17483830

[258] Ehlers GAC, Caradus JR, Fowler SV. The regulatory process and costs to seek approval for the development and release of new biological control agents in New Zealand. BioControl. 2019;65:1–12. doi:10.1007/s10526-019-09975-9.

[259] Bullock DW, Wilson WW, Neadeau JF. Genetic editing (GE) versus genetic modification (GM) in the research and development of new crop varieties: An economic comparison. Agribusiness and Applied Economics Report No. 793. Department of Agribusiness and Applied Economics. Agricultural Experiment Station, North Dakota State University, Fargo, USA. 2020 [Accessed 5 Jun 2023]. https://ageconsearch.umn.edu/record/293186

[260] Kleter GA, Kuiper HA, Kok EJ. Gene-edited crops: towards a harmonized safety assessment. Trends Biotechnol. 2019;37(5):443–47. doi:10.1016/j.tibtech.2018.11.014. 30616999

[261] Nap J-P, Metz PLJ, Escaler M, Conner AJ. The release of genetically modified crops into the environment. Plant J. 2003;33:1–18. doi:10.1046/j.0960-7412.2003.01602.x. 12943538

[262] Kumar K, Gambhir G, Dass A, Tripathi A, Singh A, Jha AK, Yadava P, Choudhary M, Rakshit S. Genetically modified crops: current status and future prospects. Planta. 2020;251: 91. Article. doi:10.1007/s00425-020-03372-8. 32236850

[263] Kuzma J, Kokotovich A. Renegotiating GM crop regulation. EMBO Rep. 2010;12:883–88. doi:10.1038/embor.2011.160. PMC3166464 21836639

[264] Jenkins D, Dobert R, Atanassova A, Pavely C. Impacts of the regulatory environment for gene editing on delivering beneficial products. In Vitro Cell Dev Biol -Plant. 2021;57:609–26. doi:10.1007/s11627-021-10201-4. 34429575 PMC8376113

[265] Kang C-K, Nicholson S. A gene-edited crop coming to a market near you: when gene-edited crops meet the grain and oilseed supply chain. RaboResearch Food And Agribusiness. 2023 [Accessed 2023 Jun 5]. https://research.rabobank.com/far/en/sectors/grains-oilseeds/a-gene-edited-crop-coming-to-a-market-near-you.html

[266] Sprink T, Eriksson D, Schiemann J, Hartung F. Regulatory hurdles for genome editing: process- vs. product-based approaches in different regulatory contexts. Plant Cell Rep. 2016;35(7):1493–506. doi:10.1007/s00299-016-1990-2. 27142995 PMC4903111

[267] Agapito-Tenfen SZ, Okoli AS, Bernstein MJ, Wikmark O-G, Myhr AI. Revisiting risk governance of GM plants: the need to consider new and emerging gene-editing techniques. Front Plant Sci. 2018;9:1874. Article. doi:10.3389/fpls.2018.01874. 30622546 PMC6308909

[268] Court of Justice of the European Union 2018. Organisms obtained by mutagenesis are GMOs and are, in principle, subject to the obligations laid down by the GMO directive., in PRESS RELEASE No 111/18, court of justice of the European Union, Luxembourg. [Accessed 2023 Jan 28]. https://curia.europa.eu/jcms/upload/docs/application/pdf/2018-07/cp180111en.pdf

[269] Callaway E. CRISPR plants now subject to tough GM laws in European Union. Nature. 2018;560(7716):16. doi:10.1038/d41586-018-05814-6. 30065322

[270] Lappin J. EU Court extends GMO directive to new plant breeding Techniques Brussels. 2018 [Accessed 2023 Jan 28]. https://gain.fas.usda.gov/Recent%20GAIN%20Publications/EU%20Court%20Extends%20GMO%20Directive%20to%20New%20Plant%20Breeding%20Techniques_Brussels%20USEU_Belgium%20EU-28_7-27-2018.pdf

[271] Arpaia S, Christiaens O, Giddings K, Jones H, Mezzetti B, Moronta-Barrios F, Perry JN, Sweet JB, Taning CNT, Smagghe G, et al. Biosafety of GM crop plants expressing dsRNA: data requirements and EU regulatory considerations. Front Plant Sci. 2020;11:940. Article. doi:10.3389/fpls.2020.00940. 32670333 PMC7327110

[272] Jorasch P. Potential, challenges, and threats for the application of new breeding techniques by the private plant breeding sector in the EU. Front Plant Sci. 2020;11:582011. Article. doi:10.3389/fpls.2020.582011. 33101349 PMC7545909

[273] Tyczewska A, Twardowski T, Woźniak-Gientka E. Agricultural biotechnology for sustainable food security. Trends Biotechnol. 2023;41(3):331–41. doi:10.1016/j.tibtech.2022.12.013. 36710131 PMC9881846

[274] Kershen DL. Sustainability council of New Zealand Trust v. The environmental protection authority: gene editing technologies and the law. GM Crops & Food. 2015;6(4):216–22. doi:10.1080/21645698.2015.1122859. 26618752 PMC5033166

[275] Fritsche S, Poovaiah C, MacRae E, Thorlby G. A New Zealand perspective on the application and regulation of gene editing. Front Plant Sci. 2018;9. Article 1323. doi:10.3389/fpls.2018.01323. PMC6144285 30258454

[276] OGTR. Department of health office of the gene technology regulator, technical review of the gene technology regulations 2001-2017-18 amendment proposals consultation, Department Of Health Office Of The Gene Technology Regulator, Australian Government. 2018 [Accessed 2023 Jan 28]. https://www.ogtr.gov.au/sites/default/files/files/2021-07/decision_ris.docx

[277] Redden R. Genetic modification for agriculture—Proposed revision of GMO regulation in Australia. Plants. 2021;10:747. Article. doi:10.3390/plants10040747. 33920391 PMC8069435

[278] Department of Biotechnology. Draft document on genome edited organisms: regulatory framework and guidelines for risk assessment. Department of Biotechnology, Ministry of Science & Technology, Government of India. 2020 [Accessed 2023 Jun 5]. https://dbtindia.gov.in/sites/default/files/DraftRegulatoryFrameworkGenomeEditing9jan2020a.pdf

[279] Sato S. Japan gives green light to genome edited waxy corn product. USDA Foreign Agricultural Service, Report Number: JA2023-0029. 2023 [Accessed 2023 Jun 5]. https://www.fas.usda.gov/data/japan-japan-gives-green-light-genome-edited-waxy-corn-product

[280] Swedish Board of Agriculture. CRISPR/Cas9 Mutated Arabidopsis. 2015 [Accessed 2023 Jan 28]. https://www.upsc.se/documents/Information_on_interpretation_on_CRISPR_Cas9_mutated_plants_Final.pdf

[281] Anon. A CRISPR definition of genetic modification. Nature Plants. 2018;4:233. doi:10.1038/s41477-018-0158-1. 29725105

[282] Parrott W. Outlaws, old laws and no laws: the prospects of gene editing for agriculture in United States. Physiol Plant. 2018;164(4):406–11. doi:10.1111/ppl.12756. 29749067

[283] Park S-H, Joung Y-H, Kim K-M, Kim J-K, Koh H-J. Gene-edited crops: present status and their future. Korean J Breed Sci. 2019;51:175–83. doi:10.9787/KJBS.2019.51.3.175.

[284] Lukanda IN, Namusoga-Kaale S, Claassen G. Media as mediators in a science-based issue: politics, foreign influence and implications on adoption of genetically modified organisms in food production in Uganda. J Sci Communication. 2023;22:A03. Article. doi:10.22323/2.22010203.

[285] Segal DJ. The promise of gene editing: so close and yet so perilously far. Front Genome Ed. 2022;4:974798. Article. doi:10.3389/fgeed.2022.974798. 35910414 PMC9334663

[286] Kuntz M. Technological risks (GMO, gene editing), what is the problem with Europe? A broader historical perspective. Frontiers In Bioeng Biotechnol. 2020;8:1308. Article. doi:10.3389/fbioe.2020.557115. PMC7681002 33240863

[287] WELL_NZ. Reframing New Zealand’s food sector opportunities. A conversation starter from Te Puna Whakaaronui: the food and fibre sector think tank. 2022 ISBN: 978-1-99-102660-6 (online). ISBN: 978-1-99-102660-6 https://fitforabetterworld.org.nz/assets/Te-Puna-Whakaaronui-publications/Reframing-New-Zealands-Food-Sector-Opportunities.pdf

[288] Atanassova A, Keiper F. Plant breeding innovation: a global regulatory perspective. Cereal Chem. 2018;95(1):8–16. doi:10.1002/cche.10021.

[289] Ahmad A, Munawar N, Khan Z, Qusmani AT, Khan SH, Jamil A, Ashraf S, Ghouri MZ, Aslam S, Mubarik MS, et al. An outlook on global regulatory landscape for genome-edited crops. Int J Mol Sci. 2021;22:11753. Article. doi:10.3390/ijms222111753. 34769204 PMC8583973

[290] Nawaz S, Kandlikar M. Drawing lines in the sand? Paths forward for triggering regulation of gene-edited crops. Sci Public Policy. 2021;48(2):246–56. doi:10.1093/scipol/scab014.

[291] Woźniak-Gientka E, Tyczewska A, Perisic M, Beniermann A, Eriksson D, Vangheluwe N, Gheysen G, Cetiner S, Abiri N, Twardowski T. Public perception of plant gene technologies worldwide in the light of food security. GM Crops & Food. 2022;13:218–41. doi:10.1080/21645698.2022.2111946. 35996854 PMC9415543

[292] Tachikawa M, Matsuo M 2023. Divergence and convergence in international regulatory policies regarding genome-edited food: how to find a middle ground. Front Plant Sci 14: 1105426 Article. 10.3389/fpls.2023.1105426 36794228 PMC9923018

[293] Gordon DR, Jaffe G, Doane M, Glaser A, Gremillion TM, Ho MD. Responsible governance of gene editing in agriculture and the environment. Nat Biotechnol. 2021;39(9):1055–57. doi:10.1038/s41587-021-01023-1. 34381209

[294] Lema MA. Regulatory aspects of gene editing in Argentina. Transgenic Res. 2019;28(Suppl 2):147–50. doi:10.1007/s11248-019-00145-2. 31321697

[295] ISAAA. (2017). Global status of commercialized biotech/GM crops in 2017: executive brief 53. Pp 153. Ithaca, NY: ISAAA. https://www.agi.gov.vn/files/files/ISAAA/ISAAA%20Brief%20No_%2053%20-%202017_compressed.pdf

[296] Dima O, Heyvaert Y, Inzé D. Interactive database of genome editing applications in crops and future policy making in the European Union. Trends Plant Sci. 2022;27(8):746–48. doi:10.1016/j.tplants.2022.05.002. 35599136

[297] Cerier S. Cracks appear in Europe’s opposition to CRISPR gene editing and other new breeding technologies. 2023 [Accessed 2023 Jun 5]. https://geneticliteracyproject.org/2023/05/16/cracks-appear-in-europes-opposition-to-crispr-gene-editing-and-other-new-breeding-technologies/

[298] European Commission. Study on the status of new genomic techniques under Union law and in light of the court of justice ruling in case C-528/16. SWD(2021) 92. 2021 [Accessed 2023 Jun 5]. https://ec.europa.eu/food/system/files/2021-04/gmo_mod-bio_ngt_exec-sum_en.pdf

[299] European Commission. Delivering the European green deal. 2021 [Accessed 2023 Jun 6]. https://commission.europa.eu/strategy-and-policy/priorities-2019-2024/european-green-deal/delivering-european-green-deal_en

[300] Kang C-K, van Rijswick C. Exploring EU regulations around gene editing – slowly but surely gee editing goes global. RaboResearch Food And Agribusiness. 2023 [Accessed 2023 Jun 6]. https://research.rabobank.com/far/en/sectors/farm-inputs/exploring-eu-regulations-around-gene-editing.html

[301] Baum CM, Kamrath C, Bröring S, De Steur H. Show me the benefits! Determinants of behavioral intentions towards CRISPR in the United States. Food Qual Prefer. 2023;107:104842. Article. doi:10.1016/j.foodqual.2023.104842.

[302] Heinemann JA, Clark K, Hiscox TC, McCabe AW, Agapito-Tenfen SZ. Are null segregants new combinations of heritable material and should they be regulated? Front Genome Ed. 2023;4. doi:10.3389/fgeed.2022.1064103. PMC9871356 36704579

[303] Kato-Nitta N, Inagaki Y, Maeda T, Tachikawa M. Effects of information on consumer attitudes towards gene-edited foods: a comparison between livestock and vegetables. CABI Agric Biosci. 2021;2:14. Article. doi:10.1186/s43170-021-00029-8.

[304] Yunes MC, Osório-Santos Z, von Keyserlingk MAG, Hötzel MJ. Gene editing for improved animal welfare and production traits in cattle: will this technology be embraced or rejected by the public? Sustainability. 2021;13:4966. Article. doi:10.3390/su13094966.

[305] Yamaguchi T. Performativity of expectations: the emergence of plant gene editing technologies in Japan. Elementa: Sci Anthropocene. 2020;8(1):1–12. doi:10.1525/elementa.036.

[306] Muhindi S. Court of Appeal upholds ban on GMOs importation. 2023 [Accessed 2023 Jun 5]. https://www.the-star.co.ke/news/2023-05-25-court-of-appeal-upholds-ban-on-gmos-importation/

[307] Applegate JS. The prometheus principle: using the precautionary principle to harmonize the regulation of genetically modified organisms. Indiana J Global Legal Stud. 2001;9:207–63. http://www.jstor.org/stable/20643826.

[308] Prakash D, Verma S, Bhatia R, Tiwary BN. Risks and precautions of genetically modified organisms. Int Scholarly Res Not. 2011;2011:369573. Article. doi:10.5402/2011/369573.

[309] Smyth SJ, Phillips PW, Kerr WA. Food security and the evaluation of risk. Global Food Secur. 2015;4:16–23. doi:10.1016/j.gfs.2014.08.001.

[310] Oxford Dictionaries 2016. Oxford dictionaries: British and World English. [Accessed 2023 May 15]. https://web.archive.org/web/20160620062539/http://www.oxforddictionaries.com/definition/english/scientific-method

[311] Margolis H. Dealing with risk: why the public and the experts disagree on environmental issues. United Kingdom: University of Chicago Press; 1997. p. 227. ISBN:9780226505299.

[312] Wiener JB. Precaution in a multi-risk world. Duke law school public law and legal theory working paper series, Working Paper No. 23. Durham, North Carolina. 2001 [Accessed 2023 Feb 26]. https://papers.ssrn.com/sol3/papers.cfm?abstract_id=293859

[313] Wiener JB. The real pattern of precaution. In: Wiener JB, Rogers MD, Hammit JK Sand P., editors. The reality of precaution: comparing risk regulation in the United States and Europe. Washington, DC: Resources for the Future Press; 2011. pp. 519–65.

[314] WTO AB. European communities - measures concerning meat and meat products, January 16, 1998 (EC- Hormones ABR). Geneva, Switzerland: World Trade Organisation; 1998.

[315] Dinneen N. Precautionary discourse: Thinking through the distinction between the precautionary principle and the precautionary approach in theory and practice. Polit Life Sci. 2013;32:2–21. doi:10.2990/32_1_2. 24047088

[316] European Commission. Communication from the commission on the pre-cautionary principle: COM (2000) 1 final. 2000 [Accessed 2023 Jan 2]. http://eur-lex.europa.eu/legal-content/EN/TXT/?uri=CELEX%3A52000DC0001

[317] Vanderzwaag DL, Fuller SD, Myers RA. Canada and the precautionary principle/approach in ocean and coastal management: wading and wandering in tricky currents. Ottawa Law Rev. 2002;34:117–58.

[318] Cartagena Protocol. Cartagena protocol on biosafety to the convention on biological diversity. 2000 [Accessed 2023 Jan 1]. https://www.cbd.int/doc/legal/cartagena-protocol-en.pdf

[319] Bourg D, Whiteside KH. Precaution and science-based environmental risk management: complementary not contradictory. In: Paleo UF, editor. Building safer communities: risk governance, spatial planning and responses to natural hazards. Lansdale, PA: IOS Press; 2009. p. 88–104.

[320] Conko G. Safety, risk and the precautionary principle: rethinking precautionary approaches to the regulation of transgenic plants. Transgenic Res. 2003;12(6):639–47. doi:10.1023/B:TRAG.0000005157.45046.8e. 14713193

[321] Aerni P Politicizing the precautionary principle: why disregarding facts should not pass for farsightedness. Front Plant Sci. 2019. 10: 1053 Article. 10.3389/fpls.2019.01053 31507627 PMC6718141

[322] Smyth SJ, Lassoued R. Agriculture R&D implications of the CJEU’s gene-specific mutagenesis ruling. Trends Biotechnol. 2019;37:337–40. doi:10.1016/j.tibtech.2018.09.004. 30293646

[323] European Commission. Directorate-general for research and innovation. ethics of genome editing. European group on ethics in science and new technologies. Opinion. 2021;(32):112. https://data.europa.eu/doi/10.2777/659034.

[324] Eichelbaum T, Allan J, Fleming J, Randerson R. Report of the royal commission on genetic modification. Report of the royal commission on genetic modification. Wellington, New Zealand. 2001 [Accessed 2023 Jan 1]. https://environment.govt.nz/assets/Publications/Files/Royal-Commission-on-GM-in-NZ-Final.pdf

[325] McGuinness Institute. An overview of genetic modification in New Zealand 1973–2013: the first forty years. Project 2058; Report 16. 2013 September [Accessed 2023 Jan 1] http://www.mcguinnessinstitute.org/project-2058-reports/

[326] Oh J, Ezezika OC 2014. To label or not to label: balancing the risks, benefits and costs of mandatory labelling of GM food in Africa. Agric & Food Secur 3: 8 Article. 10.1186/2048-7010-3-8

[327] Losey JE, Rayor LS, Carter ME. Transgenic pollen harms monarch larvae. Nature. 1999;399(6733):214. doi:10.1038/20338. 10353241

[328] Hellmich RL, Siegfried BD, Sears MK, StanleyHorn DE, Mattila HR, Spencer T, Bidne KG, Daniels MJ, Lewis LC. Monarch larvae sensitivity to Bacillus thuringiensis-purified proteins and pollen. Proc Natl Acad Sci USA. 2001;98:11925–30. doi:10.1073/pnas.211297698. 11559841 PMC59744

[329] Oberhauser KS, Prysby MD, Mattila HR, Stanley-Horn DE, Sears MK, Dively G, Olson E, Pleasants JM, Lam WK, Hellmich RL. Temporal and spatial overlap between monarch larvae and corn pollen. Proc Natl Acad Sci USA. 2001;98:11913–18. doi:10.1073/pnas.211234298. 11559838 PMC59742

[330] Ortman EE, Barry BD, Buschman LL, Calvin DW, Carpenter J, Dively GP, Foster JE, Fuller BW, Helmich RL, Higgins RA, et al. Transgenic insecticidal corn: the agronomic and ecological rationale for its use. BioScience. 2001;51:900–03. doi:10.1641/0006-3568(2001)051[0900:TICTAA]2.0.CO;2.

[331] Pleasants JM, Hellmich RL, Dively G, Sears MK, Stanley-Horn DE, Mattila HR, Foster JE, Clark PL, Jones GD. Corn pollen deposition on milkweeds in and near cornfields. Proc Natl Acad Sci USA. 2001;98:11919–24. doi:10.1073/pnas.211287498. 11559840 PMC59743

[332] Sears MK, Hellmich RL, Siegfried BD, Pleasants JM, Stanley-Horn DE, Oberhauser KS, Dively GP, Dively GP. Impact of Bt corn pollen on monarch butterfly populations: a risk assessment. Proc Natl Acad Sci USA. 2001;98:11937–42. doi:10.1073/pnas.211329998. 11559842 PMC59819

[333] Shelton AM, Sears MK. The monarch butterfly controversy: scientific interpretations of a phenomenon. Plant J. 2001;27(6):483–88. doi:10.1046/j.1365-313X.2001.01118.x. 11576433

[334] Stanley-Horn DE, Dively GP, Hellmich RL, Mattila HR, Sears MK, Rose R, Jesse LCH, Losey JE, Obrycki JJ, Lewis LC. Assessing the impact of Cry1Ab-expressing corn pollen on monarch butterfly larvae in field studies. Proc Natl Acad Sci USA. 2001;98:11931–36. https://www.pnas.org/doi/epdf/10.1073/pnas.211277798. 11559839 10.1073/pnas.211277798 PMC59745

[335] Hayes AW. The precautionary principle. Arh Hig Rada Toksikol. 2005 [Accessed 26 Feb 2023];56:161–66. https://core.ac.uk/download/pdf/14375077.pdf. 15968832

[336] Jouanin A, Boyd L, Visser RG, Smulders MJ. Development of wheat with hypoimmunogenic gluten obstructed by the gene editing policy in Europe. Front Plant Sci. 2018;9:1523. Article. doi:10.3389/fpls.2018.01523. 30405661 PMC6200864

[337] European Political Strategy Centre. Towards an innovation principle endorsed by better regulation (EPSC Strategic Notes No. Issue 14).2007 [Accessed 2023 Jan 2]. https://wayback.archive-it.org/12090/20191129102319/https://ec.europa.eu/epsc/publications/strategic-notes/towards-innovation-principle-endorsed-better-regulation_en

[338] FAO/WHO. 2006. Food safety risk analysis: A guide for national food safety authorities. FAO Food and Nutrition Paper 87. Food And Agriculture Organization Of The United Nations/World Health Organization, Rome. Pp. 50. [Accessed 2023 Jan 27]. https://apps.who.int/iris/bitstream/handle/10665/43718/9789251056042_eng.pdf 17891885

[339] FAO/WHO. 2010. FAO/WHO framework for developing national food safety emergency response plans. Food And Agriculture Organization Of The United Nations/World Health Organization, Rome. Pp. 24. [Accessed 2023 Jan 27]. https://apps.who.int/iris/handle/10665/338628

[340] Wiener JB. Precaution in a multi-risk world. In: Paustenbach DJ, editor. Human and ecological risk assessment. New York: John Wiley &c Sons; 2002. pp. 1509–31.

[341] Jonas H. The imperative of responsibility: in search of an ethics for the technological age. Chicago: University of Chicago Press; 1984. p. 255. ISBN:9780226405971.

[342] Bradford KJ, Van Deynze A, Gutterson N, Parrott W, Strauss SH. Regulating transgenic crops sensibly: lessons from plant breeding, biotechnology and genomics. Nat Biotechnol. 2005;23(4):439–44. doi:10.1038/nbt1084. 15815671

[343] Jenkins D, Juba N, Crawford B, Worthington M, Hummel A. Regulation of plants developed through new breeding techniques must ensure societal benefits. Nature Plants. 2023;9(5):679–84. doi:10.1038/s41477-023-01403-2. 37156859

[344] Camacho A, Van Deynze A, Chi-Ham C, Bennett AB. Genetically engineered crops that fly under the US regulatory radar. Nat Biotechnol. 2014;32(11):1087–91. doi:10.1038/nbt.3057. 25380439

[345] Marchant GE, Stevens YA. A new window of opportunity to reject process-based biotechnology regulation. GM Crops & Food. 2015;6(4):233–42. doi:10.1080/21645698.2015.1134406. 26930116 PMC5033199

[346] Schenkelaars P, Wesseler J. Farm-level GM coexistence policies in the EU: Context, concepts and developments. EuroChoices. 2016;15(1):5–11. doi:10.1111/1746-692X.12112.

[347] Devos Y, Demont M, Dillen K, Reheul D, Kaiser M, Sanvido O. Coexistence of genetically modified and non-GM crops in the European Union: a review. In: In: Lichtfouse E, Navarrete M, Debaeke P, Véronique S Alberola C., editors. Sustainable Agriculture. Dordrecht: Springer Netherlands; 2009. p. 203–28. doi:10.1007/978-90-481-2666-8_14.

[348] Pearsall D. GM crop co-existence. GM Crops & Food. 2013;4(3):143–50. doi:10.4161/gmcr.26303. 23988874

[349] Ramessar K, Capell T, Twyman R, Christou P. Going to ridiculous lengths—European coexistence regulations for GM crops. Nat Biotechnol. 2010;28(2):133–36. doi:10.1038/nbt0210-133. 20139947

[350] Single Vision Grains Australia. Delivering market choice with GM canola. An industry report prepared under the Single Vision Grains Australia process. 2007 [Accessed 2023 Jun 12]. http://australianoilseeds.com/__data/assets/pdf_file/0019/2935/Delivering_Market_Choice_with_GM_canola_-_FINAL_-_1MB.pdf

[351] Single Vision Grains Australia. Principles for process management of grain within the Australian supply chain: A guide for industry in an environment where GM and non-GM grain is marketed. Industry Report prepared under the Single Vision Grains Australia process. 2007 [Accessed 2023 June 12]. http://www.australianoilseeds.com/__data/assets/pdf_file/0020/2981/Principles_for_Process_Management_Final.pdf

[352] EUR-Lex. Document 32003H0556. Commission Recommendation of 23 July 2003 on guidelines for the development of national strategies and best practices to ensure the coexistence of genetically modified crops with conventional and organic farming (notified under document number C(2003) 2624). 2003 [Accessed 2023 Aug 5]. https://eur-lex.europa.eu/legal-content/EN/TXT/?uri=CELEX%3A32003H0556

[353] European Commission. COMMISSION RECOMMENDATION of 23 July 2003 on guidelines for the development of national strategies and best practices to ensure the coexistence of genetically modified crops with conventional and organic farming (notified under document number C(2003) 2624). 2003 [Accessed 2023 Aug 5]. https://faolex.fao.org/docs/pdf/eur39026.pdf

[354] Committees.parliament.uk 2014. Written evidence submitted by the Supply Chain Initiative on Modified Agricultural Crops (SCIMAC) (GMC0029). [Accessed 2023 Aug 5]. https://committees.parliament.uk/writtenevidence/50286/pdf/

[355] Rizov I, Rühl G, Langhof M, Kathage J, Rodríguez-Cerezo E. Best practice document for the coexistence of genetically modified potato with conventional and organic farming, EUR 29047. EN, Publications Office of the European Union: Luxembourg; 2018. p. 64. ISBN 978-92-79-77694-6JRC109645. doi:10.2760/055172.

[356] Giraldo PA, Shinozuka H, Spangenberg GC, Cogan NOI, Smith KF 2019. Safety assessment of genetically modified feed: Is there any difference from food? Front Plant Sci 10: 1592 Article. 10.3389/fpls.2019.01592 31921242 PMC6918800

[357] Rizov I, Cerezo ER. Best Practice Documents for coexistence of genetically modified soybean crops with conventional and organic farming. Joint Res Centre. European Coexistence Bureau – Technical Working Group for Cotton; 2016 EUR 26780 EN, ISBN 978-92-79-39542-0

[358] Loureiro I, Garcia-Ruiz E, Gutierrez E, Gomez P, Escorial MC, Chueca MC. Pollen-mediated gene flow in the cultivation of transgenic cotton under experimental field conditions in Spain. Ind Crop Prod. 2016;85:22–28. doi:10.1016/j.indcrop.2016.02.045.

[359] Van-Deynze AE, Sundstrom FJ, Bradford KJ. Pollen-mediated gene flow in California cotton depends on pollinator activity. Crop Sci. 2005;45(4):1565–70. doi:10.2135/cropsci2004.0463.

[360] Baltazar BM, Castro Espinoza L, Espinoza-Banda A, de la Fuente-Martínez JM, Garzón-Tiznado JA, González-García J, Antonio Gutiérrez M, Guzmán Rodríguez JL, Heredia Díaz O, Horak MJ, et al. Pollen-mediated gene flow in maize: implications for isolation requirements and coexistence in Mexico, the center of origin of maize. Plos One. 2015;10:e0131549. Article. doi:10.1371/journal.pone.0131549. 26162097 PMC4498909

[361] Sanvido O, Widmer F, Winzeler M, Streit B, Szerencsits E, Bigler F. Definition and feasibility of isolation distances for transgenic maize cultivation. Transgenic Res. 2008;17(3):317–35. doi:10.1007/s11248-007-9103-1. 17562214

[362] Busi R, Qin Y, Barrett-Lennard R, Powles S. Long distance pollen-mediated flow of herbicide resistance genes in *Lolium rigidum*. Theor Appl Genet. 2008;117:1281–90. doi:10.1007/s00122-008-0862-8. 18762905

[363] Wang ZY, Lawrence R, Hopkins A, Bell J, Scott M. Pollen mediated transgene flow in the wind-pollinated grass species tall fescue (*Festuca arundinacea* Schreb.). Mol Breeding. 2004;14:47–60. doi:10.1023/B:MOLB.0000037994.26287.17.

[364] Cai L, Zhou B, Guo X, Dong C, Hu X, Hou M, Liu S. Pollen-mediated gene flow in Chinese commercial fields of glufosinate-resistant canola (*Brassica napus*). Sci Bull. 2008;53:2333–41. doi:10.1007/s11434-008-0305-6.

[365] Staniland BK, McVetty PB, Friesen LF, Yarrow S, Freyssinet G, Freyssinet M. Effectiveness of border areas in confining the spread of transgenic Brassica napus pollen. Can J Plant Sci. 2000;80:521–26. doi:10.4141/P99-117.

[366] Fitzpatrick S, Reisen P, McCaslin M Pollen-mediated gene flow in alfalfa: a three-year summary of field research. In: Proceedings of the 2003 central alfalfa improvement conference, virtual meeting, 2003 July 21–25; Pp. 2. https://citeseerx.ist.psu.edu/document?repid=rep1&type=pdf&doi=a019b1990f5d5066c4185c7fe7114cb9dc60c6e4

[367] Van De Wiel CC, Groeneveld RM, Dolstra O, Kok EJ, Scholtens IM, Thissen JT, Smulders MJ, Lotz LA. Pollen-mediated gene flow in maize tested for coexistence of GM and non-GM crops in the Netherlands: effect of isolation distances between fields. NJAS: Wagening J Life Sci. 2009;56:405–23. doi:10.1016/S1573-5214(09)80007-9.

[368] Devos Y, Thas O, Cougnon M, De Clercq EM, Cordemans K, Reheul D. Feasibility of isolation perimeters for genetically modified maize. Agron Sustain Dev. 2008;28(2):195–206. doi:10.1051/agro:2007039.

[369] Milanesi J. Current and future availability of non-genetically modified soybean seeds in the USA, Brazil and Argentina. Chapter 7. In: In: Bertheau Y., editor. Genetically modified and non‐genetically modified food supply chains: co‐existence and traceability. Blackwell Publishing Ltd; 2012. pp. 89–112. 10.1002/9781118373781.ch7

[370] Ludvíková M, Griga M. Transgenic flax/linseed (Linum usitatissimum L.) - Expectations and reality. Czech J Genet Plant Breed. 2015;51:123–41. doi:10.17221/104/2015-CJGPB.

[371] Ryan CD, Smyth SJ. Economic implications of low-level presence in a zero-tolerance European import market: The case of Canadian Triffid flax. AgBioforum. 2012;15:21–30.

[372] Miles S, Ueland O, Frewer LJ. Public attitudes towards genetically-modified food. Brit Food J. 2005;107:246–62. doi:10.1108/00070700510589521.

[373] Carter C, Gruere G, McLaughlin P, MacLachlan M. California’s proposition 37: effects of mandatory labelling of GM food. Giannini Found Agric Econ, Univ California. 2012; 15(6):3–8. [Accessed 11 May 2023]. https://s.giannini.ucop.edu/uploads/giannini_public/c8/1b/c81bc863-59f2-4a08-800d-abb2c14fcc72/v15n6_2.pdf.

[374] Story D 2023. Pros and cons of mandatory GMO labeling. [Accessed 2023 Jun 6]. https://tracegains.com/blog/pros-and-cons-of-mandatory-gmo-labeling/

[375] Delgado-Zegarra J, Alvarez-Risco A, Cárdenas C, Donoso M, Moscoso S, Román BR, Del-Aguila-Arcentales S, Davies NM, Yáñez JA, Simonne A(. Labelling of Genetically Modified (GM) foods in Peru: Current dogma and insights of the regulatory and legal statutes. Int J Food Sci. 2022;2022:1–12. Article. doi:10.1155/2022/3489785. PMC9119776 35600239

[376] Chang HSC 2004. Labelling issues of organic and GM foods in Australia. Paper presented at the 48th AARES Annual Conference;[Accessed 6 Jun 2023]. Melbourne, Australia http://ageconsearch.umn.edu/record/58392/files/2004_chang.pdf

[377] Zheng Q, Wang HH. Do consumers view the genetically modified food labeling systems differently? “Contains GMO” versus “Non-GMO” labels. Chinese Econ. 2021;54(6):376–88. doi:10.1080/10971475.2021.1890356.

[378] Kim E-S. Technocratic precautionary principle: Korean risk governance of genetically modified organisms. New Genet Soc. 2014;33(2):204–24. doi:10.1080/14636778.2014.917916.

[379] Andrew J, Ismail NW, Djama M. An overview of genetically modified crop governance, issues and challenges in Malaysia. J Sci Food Agric. 2017;98:12–17. doi:10.1002/jsfa.8666. 28898466

[380] Sanmugam S, Sivakumar S, Gobalakrishnan T, Sarawanan T, Rashmi Abeweera P, Sandrasaigaran P. Perception and acceptance of genetically modified foods in Malaysia. Malaysian J Sci Adv Technol. 2021;1:144–50. doi:10.56532/mjsat.v1i4.29.

[381] Elbashir GO. Consumer protection in Islamic jurisprudence: The case of genetically modified food. J IAU Humanit Educ Sci. 2023;1:37–43. https://www.iau.edu.sa/sites/default/files/resources/consumer_protection_in_islamic_jurisprudence_the_case_of_genetically_modified_food_0.pdf.

[382] Mather D, Vikan R, Knight J. Marketplace response to GM animal products. Nat Biotechnol. 2016;34(3):236–38. doi:10.1038/nbt.3494. 26963543

[383] Vecchione M, Feldman C, Wunderlich S. Consumer knowledge and attitudes about genetically modified food products and labelling policy. Int J Food Sci Nutr. 2015;66(3):329–35. doi:10.3109/09637486.2014.986072. 25519248

[384] Codex Alimentarius. Report of the thirty fifth session of the codex committee on food labeling. 2007 [Accessed 2023 Jun 6]. http://www.fao.org/fao-who-codexalimentarius/sh-proxy/en/?lnk=1&url=https%253A%252F%252Fworkspace.fao.org%252Fsites%252Fcodex%252FShared%2BDocuments%252FArchive%252FMeetings%252FCCFL%252Fccfl35%252Ffl35_08e.pdf

[385] Pollack A. The impact of the national bioengineered food disclosure standard and its methods of disclosure on consumer preferences for gene edited and genetically modified foods. A thesis submitted to the faculty of the university of delaware in partial fulfilment of the requirements for the degree of master of science in agricultural and resource economics. 2021. [Accessed 2023 Jun 5]. https://www.proquest.com/openview/71a47e7ba937aa70c37bc25e62b81d06/1?pq-origsite=gscholar&cbl=18750&diss=y

[386] Anderson JA, Ellsworth PC, Faria JC, Head GP, Owen MDK, Pilcher CD, Shelton AM, Meissle M. Genetically engineered crops: importance of diversified integrated pest management for agricultural sustainability. Frontiers In Bioeng Biotechnol. 2019;7:24. Article. doi:10.3389/fbioe.2019.00024. PMC6391707 30842944

[387] Clark LF. Framing the uncertainty of risk: models of governance for genetically modified foods. Sci Public Policy. 2013;40(4):479–91. doi:10.1093/scipol/sct001.

[388] Ruder S-L, Kandlikar M. Governing gene-edited crops: risks, regulations, and responsibilities as perceived by agricultural genomics experts in Canada. J Responsible Innovation. 2023;10(1). doi:10.1080/23299460.2023.2167572.

[389] Eriksson D, Harwood W, Hofvander P, Jones H, Rogowsky P, Stöger E, Visser RG. A welcome proposal to amend the GMO legislation of the EU. Trends Biotechnol. 2018;36(11):1100–03. doi:10.1016/j.tibtech.2018.05.001. 29807731 PMC6198110

[390] Neal T. The Regional Governance of Genetically Modified Crops: What Does the Future Hold for ECOWAS?. Africa Policy Journal – A Harvard Kennedy School Student Publication. 2019 [Accessed 2023 Jan 29]. https://apj.hkspublications.org/the-regional-governance-of-gmos-ecowas/

[391] Rock JS, Schnurr MA, Kingiri A, Glover D, Stone GD, Ely A, Fischer K. Beyond the genome: genetically modified crops in africa and the implications for genome editing. Dev Change. 2023;54(1):117–42. doi:10.1111/dech.12750.

[392] Ishii T, Araki M. Consumer acceptance of food crops developed by genome editing. Plant Cell Rep. 2016;35(7):1507–18. doi:10.1007/s00299-016-1974-2. 27038939

[393] Parvaiz A, Munawa S, Nawaz MA, Mustafa G, Khan MS, Joyia FA. The need of regulations for GM crops and products thereof. In: In: Nawaz MA, Chung G, Golokhvast KS Tsatsakis AM., editors. Gmos and Political Stance. Academic Press; 2023. p. 15–30. 10.1016/B978-0-12-823903-2.00010-X

[394] Schmidt SM, Belisle M, Frommer WB. The evolving landscape around genome editing in agriculture: Many countries have exempted or move to exemt forms of genome editing from GMO regulation of crop plants. EMBO Reports. 2020;21(6): e50680. doi:10.15252/embr.202050680. PMC7271327 32431018

